# *ZMAT3* hypomethylation contributes to early senescence of preadipocytes from healthy first‐degree relatives of type 2 diabetics

**DOI:** 10.1111/acel.13557

**Published:** 2022-02-11

**Authors:** Rosa Spinelli, Pasqualina Florese, Luca Parrillo, Federica Zatterale, Michele Longo, Vittoria D’Esposito, Antonella Desiderio, Annika Nerstedt, Birgit Gustafson, Pietro Formisano, Claudia Miele, Gregory Alexander Raciti, Raffaele Napoli, Ulf Smith, Francesco Beguinot

**Affiliations:** ^1^ Department of Translational Medical Sciences Federico II University of Naples Naples Italy; ^2^ URT Genomics of Diabetes Institute of Experimental Endocrinology and Oncology National Research Council Naples Italy; ^3^ Lundberg Laboratory for Diabetes Research Department of Molecular and Clinical Medicine Sahlgrenska Academy University of Gothenburg Gothenburg Sweden

**Keywords:** adipose precursor cells, aging, cellular senescence, DNA methylation, first‐degree relatives of type 2 diabetics, senolytics, type 2 diabetes, *ZMAT3*

## Abstract

Senescence of adipose precursor cells (APC) impairs adipogenesis, contributes to the age‐related subcutaneous adipose tissue (SAT) dysfunction, and increases risk of type 2 diabetes (T2D). First‐degree relatives of T2D individuals (FDR) feature restricted adipogenesis, reflecting the detrimental effects of APC senescence earlier in life and rendering FDR more vulnerable to T2D. Epigenetics may contribute to these abnormalities but the underlying mechanisms remain unclear. In previous methylome comparison in APC from FDR and individuals with no diabetes familiarity (CTRL), *ZMAT3* emerged as one of the top‐ranked senescence‐related genes featuring hypomethylation in FDR and associated with T2D risk. Here, we investigated whether and how DNA methylation changes at *ZMAT3* promote early APC senescence. APC from FDR individuals revealed increases in multiple senescence markers compared to CTRL. Senescence in these cells was accompanied by *ZMAT3* hypomethylation, which caused *ZMAT3* upregulation. Demethylation at this gene in CTRL APC led to increased *ZMAT3* expression and premature senescence, which were reverted by *ZMAT3* siRNA. Furthermore, *ZMAT3* overexpression in APC determined senescence and activation of the p53/p21 pathway, as observed in FDR APC. Adipogenesis was also inhibited in *ZMAT3*‐overexpressing APC. In FDR APC, rescue of *ZMAT3* methylation through senolytic exposure simultaneously downregulated *ZMAT3* expression and improved adipogenesis. Interestingly, in human SAT, aging and T2D were associated with significantly increased expression of both *ZMAT3* and the *P53* senescence marker. Thus, DNA hypomethylation causes *ZMAT3* upregulation in FDR APC accompanied by acquisition of the senescence phenotype and impaired adipogenesis, which may contribute to FDR predisposition for T2D.

## Graphical abstract

The senescence of adipose precursor cells (APC) contributes to the increased risk of Type 2 Diabetes (T2D) in both elderly subjects and young and non‐obese first‐degree relatives of T2D patients (FDR). DNA hypomethylation in the APC from FDR individuals leads to an upregulation of ZMAT3 which activates the p53/p21 pathway, causing the acquisition of the senescence phenotype and impaired adipogenesis in these progenitor cells.

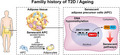

**Abbreviations**
APC: Adipose precursor cells
AT: Adipose tissue
CTRL: Individuals with no diabetes familiarity
DMR: Differentially methylated region
FDR: First‐degree relatives of T2D individuals
IR: Insulin resistance
SAT: Subcutaneous adipose tissue
SASP: Senescence‐associated secretory phenotype
SNC: Senescent cells
T2D: Type 2 diabetes

## 1 INTRODUCTION

Over the past decades, medical advances led to improved quality of life and to extended life expectancy. However, this positive trend has also been associated with a marked increase in the prevalence of age‐related disorders, including type 2 diabetes (T2D) (Kirkman et al., 2012). While T2D is common in the elderly population, its prevalence among the youth is presently rising (Lascar et al., 2018). Thus, T2D is now considered a major threat to human health worldwide, making the need to achieve a better understanding of its impact on human health a priority (Unnikrishnan et al., 2017).

Similar to aging, obesity and family history of T2D are major predisposing risk factors for the development of T2D (InterAct Consortium et al., 2013; Kirkman et al., 2012). Indeed, both obese subjects and first‐degree relatives of type 2 diabetics (FDR) exhibit premature onset of molecular abnormalities associated with increased risk of T2D, mimicking accelerated aging. It is possible that aging, obesity, and T2D familiarity share common mechanisms underlying T2D pathogenesis (Spinelli et al., 2020). Aging and obesity are associated with an increased burden of senescent cells (SNC) at tissues of relevance to T2D development (Khosla et al., 2020). SNC are characterized by irreversible growth arrest, loss of differentiation capacity, and acquisition of senescence‐associated secretory phenotype (SASP) which includes a wide range of proinflammatory cytokines and chemokines, proteases, and growth factors (Herranz & Gil, 2018). Likely owing to their SASP, a small number of SNC may disrupt tissue homeostasis and interfere with organ function through both cell‐autonomous and paracrine mechanisms (Tchkonia & Kirkland, 2018). Accumulation of SNC in the subcutaneous adipose tissue (SAT) is identified as a major cause of the age‐ and obesity‐related inflammation and metabolic derangement, which are responsible for T2D development (Spinelli et al., 2020). In this tissue, adipose precursor cells (APC) are among the more senescent‐susceptible progenitor cells (Wissler Gerdes et al., 2020). Also, senescent APC are unable to differentiate into fully functional and insulin‐responsive adipocytes, reducing the ability of the SAT to expand when challenged with nutrient excess (Gustafson et al., 2019). As a consequence, the SAT becomes hypertrophic, inflamed, and dysfunctional, and excess lipids accumulate in visceral fat and ectopic tissues (Smith & Kahn, 2016). These events drive insulin resistance (IR) and low‐grade inflammation and predispose for T2D (Acosta et al., 2016). Consistently, transplanting senescent APC into young animals causes IR (Xu et al., 2018). On the contrary, senolytic clearance of senescent APC from old or obese mice reduces adipocyte hypertrophy, promotes adipogenesis, and ameliorates the inflammation and IR (Palmer et al., 2019). Even when lean, FDR individuals feature SAT dysfunction and inflammation accompanied by adipocyte hypertrophy, due to impaired differentiation of resident APC which render these subjects vulnerable to T2D development (Arner et al., 2011; Henninger et al., 2014). Interestingly, a recent investigation reported that, in individuals who are FDR, the reduced ability of subcutaneous APC to differentiate is associated with impaired ability to suppress the p53 senescence marker after adipocyte differentiation (Gustafson et al., 2019), suggesting that SAT abnormalities related to T2D familiarity reflect the detrimental effects of early APC senescence.

Despite intensive investigation, the molecular mechanisms leading to APC senescence and their pathophysiological relevance in T2D remain unclear. Epigenetic modifications, in particular DNA methylation, are emerging as key players in these events and growing evidence now points to relevance of DNA hypomethylation as a senescence inducer (Atkinson & Keith, 2007; Cheng et al., 2017). Interestingly, we demonstrated that the epigenetic signature of subcutaneous APC in FDR subjects is characterized by a number of DNA hypomethylation events (Parrillo et al., 2020). Thus, the hypothesis that DNA methylation level determines occurrence of an early senescence phenotype in APC of FDR individuals and contributes to their predisposition toward T2D, deserves to be investigated.

To gain mechanistic insights into this issue, this study has integrated data from our previous characterization of the FDR methylome (Parrillo et al., 2020) and the SeneQuest public database (Gorgoulis et al., 2019). The work revealed that several hypomethylated genes identified in APC from FDR subjects are also related to senescence. We then focused on the *Zinc Finger Matrin*‐*Type 3* (*ZMAT3*) as this gene emerged as one of the top‐ranked senescence‐related genes which also feature hypomethylation in individuals who are FDR of T2D patients. ZMAT3 is a positive regulator of p53 which in turn acts as a molecular link between pathways involved in cell senescence, inflammation, and IR in the adipose tissue (AT) (Minamino et al., 2009; Vilborg et al., 2009). We now show that *ZMAT3* hypomethylation contributes to the early APC senescence occurring in FDR and represents an epigenetic signature of these cells.

## 2 RESULTS

### 2.1 Senescence markers in APC from FDR of T2D subjects

**FIGURE 1:**
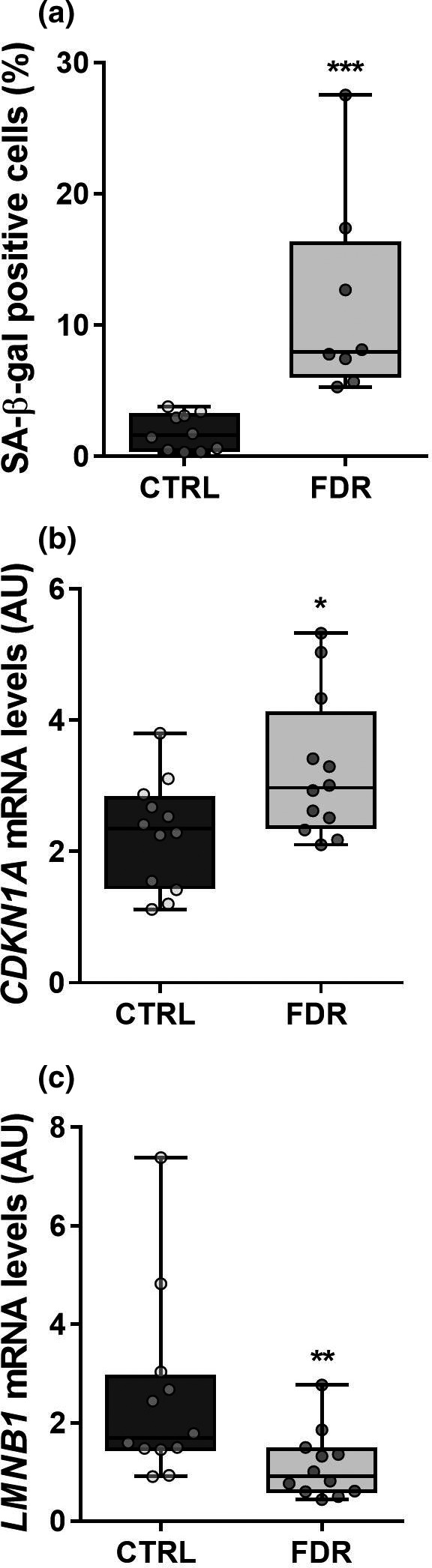
Senescence markers in APC from FDR and CTRL. (a) Flow cytometric detection of the SA‐β‐gal‐positive cells in APC from FDR (*n *= 8) and CTRL (*n *= 10) subjects available from our study cohort. Values are presented as percentage (%). The mRNA levels of *CDKN1A* (b) and *LMNB1* (c) were measured by qPCR and normalized to *RPL13A* expression in APC from FDR (*n *= 12) and CTRL (*n *= 12) subjects. Values are presented as absolute units (AU). (a‐c) Data are shown as boxplots (min‐max) with all individual values. Significance was determined by Mann–Whitney test (a,c) or unpaired Student's *t*‐test (b). *p < 0.05, **p < 0.01, ***p < 0.001 versus CTRL

To explore the hypothesis that the early senescence of APC occurs in individuals at high risk for developing T2D, we have first compared senescence markers in APC from first‐degree relatives of T2D patients (FDR) and subjects with no familiarity for diabetes (CTRL). We have analyzed a panel of senescence markers in these cells, as none of them features absolute specificity (Wiley et al., 2017). A threefold increase in the percentage of senescence‐associated beta‐galactosidase (SA‐β‐gal)‐positive APC was observed in the FDR compared to the CTRL (Figure 1a). Based on flow cytometric forward scatter and side scatter evaluation, this difference was accompanied, respectively, by enlarged size and increased cytoplasmic granularity in the FDR APC (Figure S1), along with upregulation of the p21‐encoding *CDKN1A* gene (Figure 1b) and a consistent increase in the percentage of cells arrested in the G_1_ phase of cell cycle (Figure S2a). Accordingly, FDR APC showed a significant slower growth rate compared to controls (Figure S2b). The *Lamin B1* (*LMNB1*) mRNA levels were also reduced in the FDR APC (Figure 1c). Subsequently, to examine whether the APC from FDR subjects also release SASP factors we compared conditioned media (CM) obtained from FDR and CTRL cells maintained in culture for 24 h. As presented in Table 1, several key SASP factors were present at higher levels in the media conditioned by FDR as compared to CTRL APC. These include interleukin 6 (IL6), monocyte chemotactic protein 1 (MCP1), regulated on activation normal T‐cell‐expressed and T‐cell‐secreted (RANTES), interleukin 8 (IL8), and macrophage inflammatory protein 1 beta (MIP1b). Consistently, increased mRNA expression of *IL6*, *MCP1*, and *RANTES* was detected in APC from individuals who were FDR (data not shown).

**TABLE 1:** SASP factor protein levels in the media conditioned by FDR and CTRL APC

Variables	FDR APC	CTRL APC	*p* value
IL6 (pg/ml/10^5^ cells)	1099.0 (809.1; 1171.0)	193.0 (60.2; 633.0)	*0.0021*
MCP1 (pg/ml/10^5^ cells)	118.0 (82.2; 245.0)	42.0 (12.4; 117.1)	*0.0430*
RANTES (pg/ml/10^5^ cells)	6.4 (2.6; 7.7)	1.6 (0.3; 3.0)	*0.0165*
IL8 (pg/ml/10^5^ cells)	112.0 (49.3; 340.6)	30.0 (15.7; 60.7)	*0.0260*
MIP1b (pg/ml/10^5^ cells)	1.0 (1.8;3.8)	0.9 (0.3; 1.9)	*0.0322*
EOTAXIN (pg/ml/10^5^ cells)	0.5 (0.3; 2.3)	0.4 (0.1; 1.0)	0.2735
FGF (pg/ml/10^5^ cells)	5.9 (4.6; 7.3)	3.9 (1.8; 6.2)	0.1522
G‐CSF (pg/ml/10^5^ cells)	24.5 (15.8; 36.1)	7.4 (4.3; 90.5)	0.3042
IFNɣ (pg/ml/10^5^ cells)	5.7 (3.4; 6.1)	3.9 (0.9; 7.7)	0.2370
VEGF (pg/ml/10^5^ cells)	80.3 (29.5; 135.3)	55.4 (30.7; 140.9)	0.6170
TNFα (pg/ml/10^5^ cells)	1.7 (0.7; 2.7)	0.7 (0.1; 2.6)	0.2646
IL15 (pg/ml/10^5^ cells)	68.5 (43.7; 94.7)	37.0 (23.5; 72.1)	0.1220

Note

SASP factor protein levels in the media conditioned by FDR (*n*=8) and CTRL (*n*=10) APC were measured by multiplex assay and normalized by cell number. Detectable APC‐released SASP factors are reported. Results are shown as median (first quartile‐Q1; third quartile‐Q3) and compared between groups using Mann–Whitney test. *p* value versus CTRL APC. *p *≤ 0.05 was considered statistically significant and it is italicised in the table.

FDR, first‐degree relatives of T2D subjects; CTRL, subjects with no diabetes familiarity; APC, adipose precursor cells; IL, interleukin; MCP1, monocyte chemotactic protein 1; RANTES, regulated on activation normal T‐cell‐expressed and T‐cell‐secreted; MIP1b, macrophage inflammatory protein 1 beta; FGF, fibroblast growth factor; G‐CSF, granulocyte‐colony stimulating factor; IFNɣ, interferon gamma; VEGF, vascular endothelial cell growth factor; TNFα, tumor necrosis factor alpha.

Thus, APC from subjects who are FDR feature increased marks of senescence as compared to individuals with no familiarity for diabetes. Since these cells also acquire a distinctive SASP, their presence may likely impact the surrounding AT.

### 2.2 The role of *ZMAT3* in early senescence of FDR APC

**FIGURE 2:**
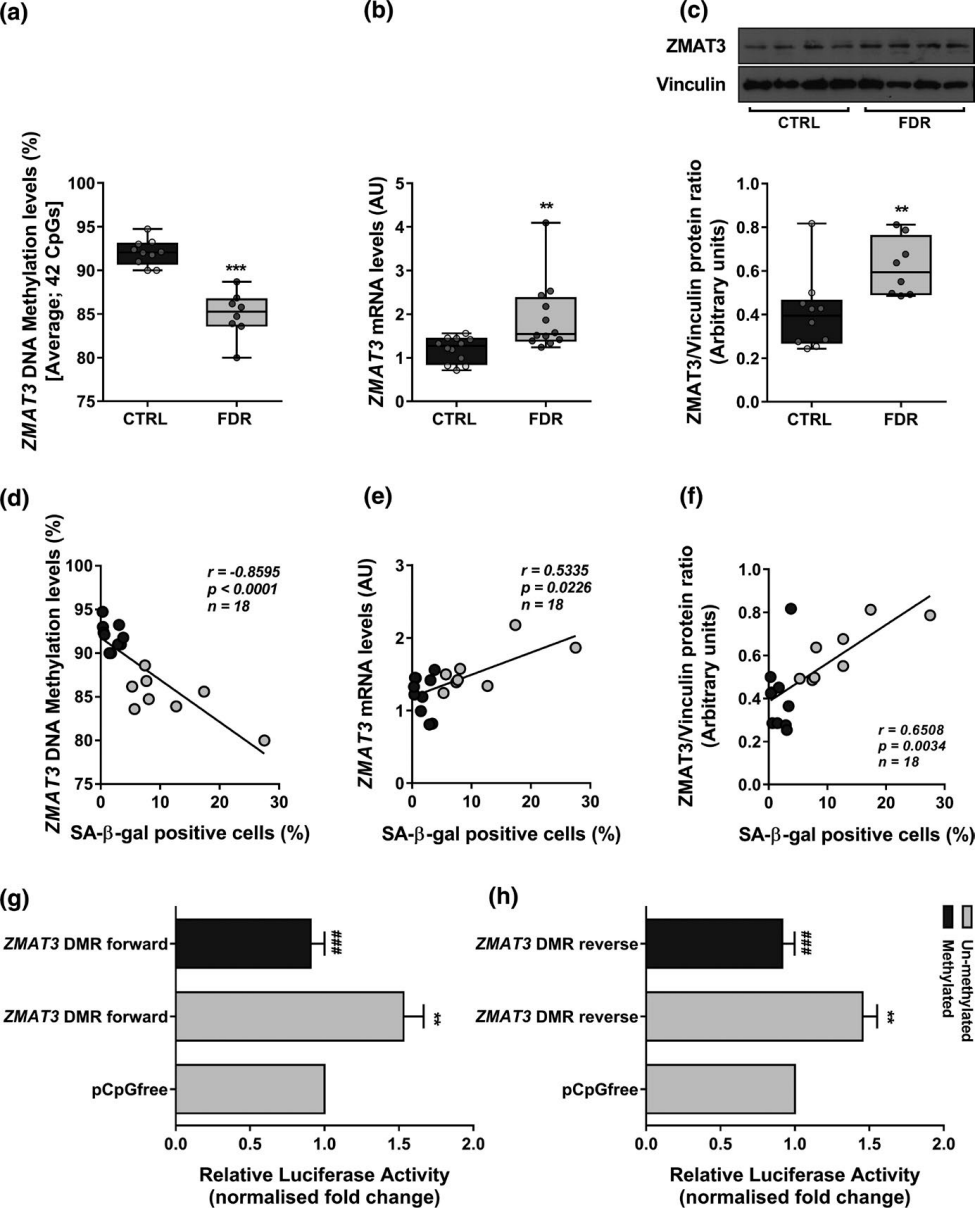
DNA methylation and expression of *ZMAT3* in APC from FDR and CTRL. (a) Changes in average DNA methylation levels at 42 CpGs within the *ZMAT3* DMR were detected by bisulfite sequencing (BS) in APC from FDR (*n *= 8) and CTRL (*n *= 10) subjects available from our study cohort. (b) The *ZMAT3* mRNA levels were measured by qPCR and normalized to *RPL13A* expression in APC from FDR (*n *= 12) and CTRL (*n *= 12) subjects. Data are presented as absolute units (AU). (c) The ZMAT3 protein levels were assessed by Western blot in APC from FDR (*n *= 8) and CTRL (*n *= 10) subjects available from our study cohort. Vinculin served as a loading control. The upper figure shows representative blots; the lower figure shows result quantitation. (a‐c) Data are shown as boxplots (min‐max) with all individual values. Significance was determined by unpaired Student's *t*‐test (a) or Mann–Whitney test (b,c). **p < 0.01, ***p < 0.001 *vs* CTRL. (d‐f) Correlation between the percentage of SA‐β‐gal‐positive cells and *ZMAT3* DNA methylation or mRNA or protein levels in the same APC samples from FDR and CTRL. Spearman's correlation coefficient *r*, *p* value, and number of samples (*n*) are indicated in the graph. Dark gray circles represent CTRL; light gray circles represent FDR. (g,h) The *ZMAT3* DMR was cloned into a luciferase reporter vector devoid of CpGs in both forward (*ZMAT3* DMR forward) and reverse (*ZMAT3* DMR reverse) orientations. These constructs were either methylated or mock‐treated (un‐methylated). The results were normalized using a co‐transfected renilla luciferase control vector and are presented as fold change relative to the mock‐treated empty vector (pCpGfree). Data are shown as mean ±SEM of three independent experiments. Significance was determined by one‐way repeated measures ANOVA followed by Tukey's multi‐comparison test. **p < 0.01 versus pCpGfree; *^###^p *< 0.001 *vs* un‐methylated *ZMAT3* DMR forward (g) or un‐methylated *ZMAT3* DMR reverse (h)

To gain novel insight into the potential mechanisms responsible for the senescence phenotype exhibited by the APC from FDR subjects, we focused on the RNA‐binding protein ZMAT3 as its role in senescence, aging and age‐associated diseases is well‐established (Dong et al., 2018; Kim et al., 2017). In addition, our previous MeDIP‐Seq study in APC from these same FDR subjects (Parrillo et al., 2020) has identified a CpG island in the intronic *chr3*:*179032279*–*179033001* region at the *ZMAT3* gene showing significant hypomethylation compared to CTRL individuals, providing a potential basis for altered *ZMAT3* gene transcription in FDR. To address this issue, direct testing by bisulfite sequencing was performed, which confirmed that DNA methylation level at this *ZMAT3* region is significantly reduced in FDR compared to CTRL APC (Figure 2a). Consistently, as shown in Figure 2b,c, we found enhanced *ZMAT3* expression in the FDR subjects, both at mRNA and protein levels. The SA‐β‐gal senescence marker strongly correlated with *ZMAT3* DNA methylation, mRNA, and protein levels in both FDR and CTRL subjects (Figure 2d‐f). Altogether, these findings raised the possibility that epigenetic dysregulation of *ZMAT3* is involved in the early APC senescence observed in subjects who are FDR of T2D patients.

### 2.3 Functional analysis of the *ZMAT3* intronic region

To confirm the regulatory role of CpG island in the intronic *chr3*:*179032279*–*179033001 ZMAT3* region, we have cloned the region in both the forward and reverse orientations in a luciferase reporter vector. The constructs were either *in vitro* methylated or mock‐treated (un‐methylated) and then transfected in HEK‐293 cells. Subsequent analysis demonstrated that the un‐methylated construct harboring the *ZMAT3* intronic differentially methylated region (DMR) featured higher luciferase activity compared to the mock‐treated empty vector, in both the forward and reverse orientations (Figure 2g,h). Importantly, both these constructs displayed reduced transcriptional activity upon being *in vitro* methylated (Figure 2g,h), indicating that the intronic DMR functions as a DNA methylation‐sensitive region regulating *ZMAT3* transcription.

To further test whether, in the APC, methylation‐induced changes in *ZMAT3* transcription associate with premature senescence in APC, we have exposed the cells from CTRL subjects to 5‐azacytidine (5‐AZA). This hypomethylating agent has been previously shown to induce senescence in both human primary cells and cancer cell lines (So et al., 2011). After treatment with 10 μM 5‐AZA for 72 h, more than 10% of APC became positive for SA‐β‐gal (Figure S3a). The effect of 5‐AZA on these cells was similarly sized as that of the senescence inducer hydrogen peroxide which was used as positive control for stress‐induced premature senescence (Chen et al., 2007). Interestingly, in 5‐AZA‐treated APC, appearance of senescence was paralleled by decreased DNA methylation at the *ZMAT3* intronic DMR (Figure S3b) and by upregulation of the *ZMAT3* expression (Figure S3c,d), supporting a causal link between epigenetic dysregulation of *ZMAT3* and early APC senescence. To prove that senescence induced by 5‐AZA exposure is caused, at least in part, by upregulation of *ZMAT3*, we knocked down its expression in CTRL APC, followed by 5‐AZA treatment. As shown in Figure S4, a higher percentage of senescent cells was detected following the exposure to 5‐AZA. This effect was significantly reduced by siRNA‐mediated knock‐down of *ZMAT3*, strengthening the interpretation that reduced *ZMAT3* methylation increases its expression and causes premature senescence in APC.

### 2.4 Senescence induction by *ZMAT3* in human APC

**FIGURE 3:**
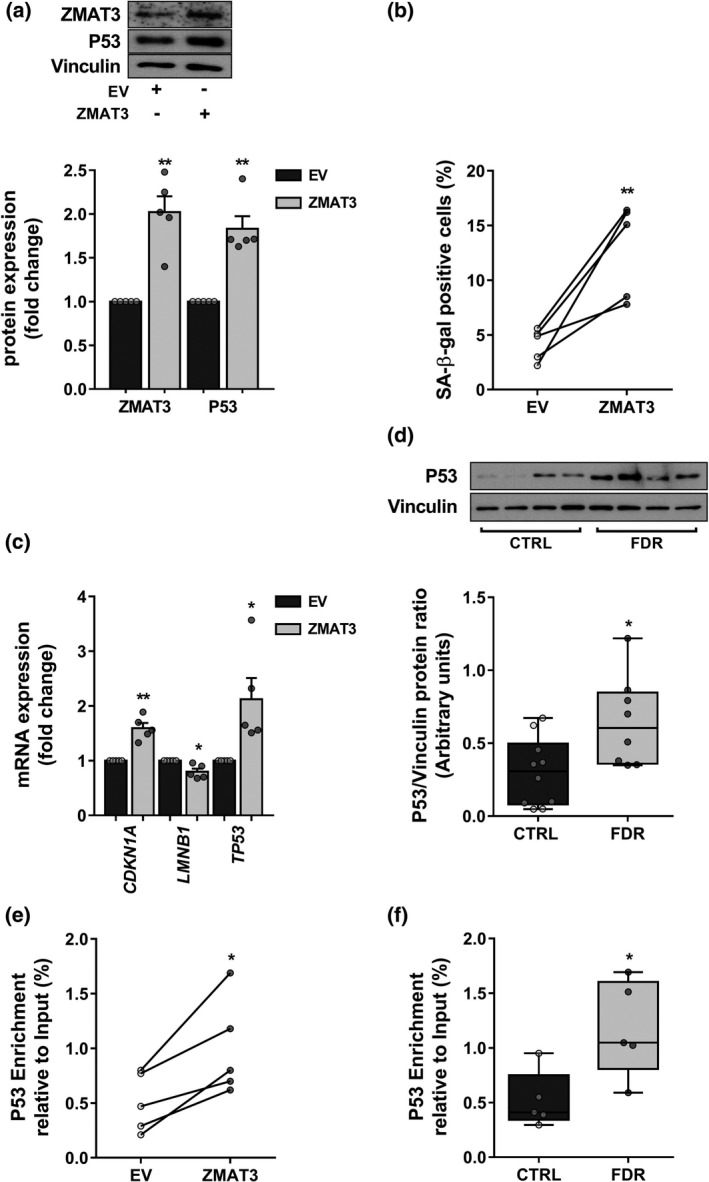
Mechanism of senescence in *ZMAT3*‐overexpressing APC. (a‐c,e) Five biologically independent APC samples randomly selected in the CTRL group were transfected with the pCMV6‐*ZMAT3* expression vector (ZMAT3) or the empty vector (EV). (a) The fold change of ZMAT3 and P53 proteins was assessed by Western blot in ZMAT3‐ *vs* EV‐transfected APC. Vinculin served as a loading control. The upper figure shows representative blots; the lower figure shows result quantitation. (b) Flow cytometric detection of the SA‐β‐gal‐positive cells in ZMAT3‐ or EV‐transfected APC. Values are presented as percentage (%). (c) mRNA levels of *CDKN1A*, *LMNB1*, and *TP53* were measured by qPCR and are presented as fold change in figure. mRNA expression was normalized first to *28S* and then to expression in EV‐transfected APC. (d) The P53 protein levels were assessed by Western blot in APC from FDR (*n *= 8) and CTRL (*n *= 10) subjects available from our study cohort. Vinculin served as a loading control. The upper figure shows representative blots; the lower figure shows result quantitation. (e,f) ChIP analysis for P53 binding at the *CDKN1A* promoter region containing the validated P53 RE in ZMAT3‐ or EV‐transfected APC and APC from FDR (*n *= 5) and CTRL (*n *= 5) subjects randomly selected in each study group. Results are expressed as percent enrichment relative to input DNA. (a,c) Data are shown as mean ±SEM. Dots represent individual level data. (b,e) Data are shown as scatterplot with lines joining paired points. (a‐c,e) Significance was determined by paired Student's *t*‐test. *p < 0.05, **p < 0.01 *vs* EV. (d,f) Data are shown as boxplots (min‐max) with all individual values. Significance was determined by unpaired Student's *t*‐test. *p < 0.05 versus CTRL

To directly demonstrate whether *ZMAT3* enhanced expression causes senescence in human preadipocytes, we transfected the pCMV6‐*ZMAT3* construct in APC from donors with no diabetes familiarity. This procedure determined a twofold increase in the ZMAT3 protein levels upon 72 h from transfection (Figure 3a). This increase was of similar size as that occurring in APC from FDR subjects and sufficient to trigger prompt cell senescence, as indicated by a threefold increase in the percentage of SA‐β‐gal‐positive APC (Figure 3b). Consistently, mRNA levels of *CDKN1A* were increased while that of *LMNB1* were significantly reduced in the cells transfected with the pCMV6‐*ZMAT3* construct (Figure 3c). The levels of the major SASP components IL6 (mRNA and protein), MCP1 (mRNA and protein), and MIP1b (protein) were also significantly enhanced in these cells (Table S1; Figure S5), identifying a SASP pattern in *ZMAT3*‐overexpressing APC similar to that observed in APC from FDR subjects.

Next, we sought to elucidate the molecular mechanism by which *ZMAT3* induces senescence in APC. ZMAT3 is known to block *TP53* mRNA deadenylation, resulting in *TP53* mRNA stabilization and thus enhanced P53 protein expression (Vilborg et al., 2009). Interestingly, *ZMAT3* upregulation by 5‐AZA was accompanied by an increase in P53 expression and reverted by siRNA‐mediated *ZMAT3* silencing (Figure S6). Furthermore, overexpression of *ZMAT3* in APC from CTRL donors caused a ~twofold increase in the P53 mRNA and protein levels (Figure 3a,c), supporting the hypothesis that *ZMAT3* mediated APC senescence through P53. Interestingly, a significant upregulation of P53 was detected in FDR APC compared to CTRL APC (Figure 3d; Figure S7). The protein levels of ZMAT3 significantly correlated with the P53 mRNA and protein levels in the same APC samples from FDR and CTRL subjects (Figure S8a,b), revealing upregulation of P53 by ZMAT3 also in FDR individuals. The finding that the mRNA levels of the p21‐encoding *CDKN1A* gene, a pro‐senescence mediator of p53, increased in both *ZMAT3*‐overexpressing APC and FDR APC, prompted us to investigate the possibility that *ZMAT3* acts through the p53/p21 pathway. To explore this further hypothesis, we examined whether upregulation of ZMAT3 causes activation of a validated P53 response element (RE) in the upstream regulatory region of the *CDKN1A* promoter (el‐Deiry et al., 1993; Laptenko et al., 2011). To this end, we cloned the *CDKN1A* promoter region (−2297 to −2207 bp upstream the TSS) containing the P53 RE (−2281 to −2261 bp upstream the TSS) in a reporter gene plasmid to perform luciferase assays. Also, the *CDKN1A* reporter construct was mutagenized at the invariant G/C basepairs within the P53 RE, to prevent P53 from binding to this nucleotide sequence (Figure S9a). The wild‐type or mutagenized *CDKN1A* reporter construct was transfected in APC from CTRL donors, and promoter activity was first assessed in the absence or presence of the pCMV6‐*TP53* expression vector (Figure S9b). P53 overexpression significantly enhanced the luciferase activity of the wild‐type *CDKN1A* reporter construct but did not affect that of the mutagenized *CDKN1A* reporter construct (Figure S9c), confirming that P53‐dependent transactivation of the *CDKN1A* promoter was effectively prevented by point mutations in the P53 RE. Subsequently, the wild‐type or the mutated *CDKN1A* reporter construct was co‐transfected in CTRL APC with the pCMV6‐*ZMAT3* expression vector. P53 upregulation following *ZMAT3* overexpression (Figure S9d) resulted in increased luciferase activity of the wild‐type *CDKN1A* reporter construct, while this effect was completely lost when the P53 RE in the *CDKN1A* reporter construct was mutagenized (Figure S9e), indicating that *ZMAT3* promotes the transcriptional activation of *CDKN1A* by P53. To strengthen this finding, we evaluated the P53 occupancy at the same *CDKN1A* promoter region in *ZMAT3*‐overexpressing APC. Chromatin immunoprecipitation (ChIP) experiments revealed that *ZMAT3* overexpression elicited a significant enrichment of P53 in the *CDKN1A* promoter (Figure 3e), confirming that ZMAT3 regulates *p21* expression at the transcriptional level by increasing the capability of P53 to bind its RE within the *CDKN1A* promoter. Consistently, we also found a twofold increase in P53 abundance at the *CDKN1A* promoter in the FDR compared to the CTRL APC (Figure 3f). Interestingly, the P53 binding at the *CDKN1A* promoter positively correlated with the *CDKN1A* mRNA levels in the same APC samples from FDR and CTRL subjects (Figure S10), supporting that transcription of *CDKN1A* in FDR APC was enhanced by P53.

To confirm that activation of the p53/p21 pathway is necessary for the induction of senescence by ZMAT3, we have blocked the transcriptional activity of P53 using the specific inhibitor pifithrin alpha (PFTα) (Sohn et al., 2009). To this end, we transfected the pCMV6‐*ZMAT3* construct in APC from subjects with no diabetes familiarity in the presence or the absence of 50 nM PFTα. As shown in Figure S11, the levels of *CDKN1A* mRNA and the percentage of SA‐β‐gal‐positive cells were significantly lower in the presence of PFTα, indicating that the p53/p21 pathway plays a major role in the *ZMAT3*‐induced senescence in APC. Taken together, these results indicated that *ZMAT3* causes APC senescence through the p53/p21 pathway.

### 2.5 Adipocyte differentiation of *ZMAT3*‐overexpressing APC

**FIGURE 4:**
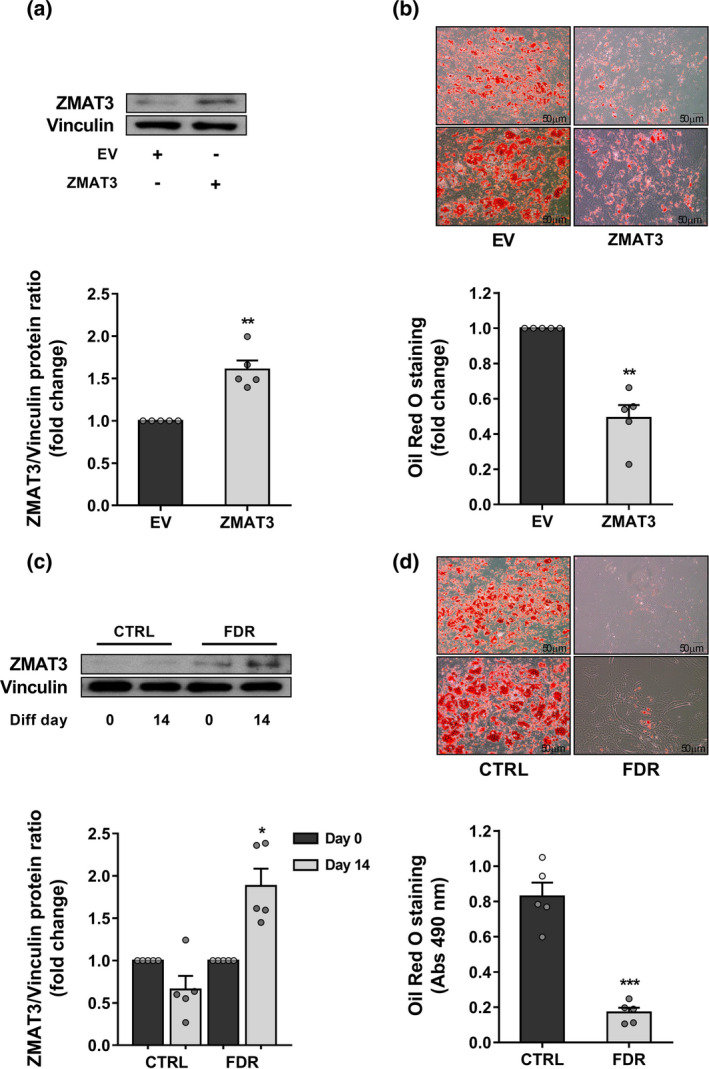
*ZMAT3* upregulation in APC is linked to impaired adipogenesis. (a,b) APC from CTRL donors (*n*=5) were transfected with the pCMV6‐*ZMAT3* expression vector (ZMAT3) or the empty vector (EV) and differentiated for 15 days. (a) *ZMAT3* overexpression was confirmed by Western blot in ZMAT3‐ *vs* EV‐transfected APC at differentiation day 15. The upper figure shows representative blots; the lower figure shows result quantitation. (b) Oil Red O staining was used to assess the degree of differentiation. Representative microphotographs showing lipid accumulation of *ZMAT3*‐ (right) and EV‐transfected (left) APC at differentiation day 15. Upper panel at 10x magnification; bottom panel at 20x magnification. Scale bar 50 μm. Bar graph shows photometric quantification of Oil Red O staining measured at 490 nm. Results are normalized to the absorbance in EV‐transfected APC. (a,b) Data are shown as mean ±SEM of five biologically independent APC samples randomly selected in the CTRL group. Dots represent individual level data. Significance was determined by paired Student's *t*‐test. **p < 0.01 versus EV. (c,d) APC from FDR (*n *= 5) and CTRL (*n*=5) subjects randomly selected in each study group were differentiated for 15 days. (c) The fold change of ZMAT3 protein was measured by Western blot in differentiated APC (diff. day 15) *vs* APC before adipogenic induction (diff. day 0) in each group. The upper figure shows representative blots; the lower figure shows result quantitation. Data are shown as mean ±*SEM*. Dots represent individual level data. Significance was determined by paired Student's *t*‐test. *p < 0.05 versus APC at diff. day 0. (d) Oil Red O staining of differentiated APC from FDR and CTRL. Representative microphotographs showing lipid accumulation of APC from FDR (right) and CTRL (left) at diff. day 15. Upper panel at 10× magnification; bottom panel at 20× magnification. Scale bar 50 μm. Bar graph shows photometric quantification of Oil red O staining measured at 490 nm. Data are shown as mean ±SEM. Dots represent individual level data. Significance was determined by unpaired Student's *t*‐test. ***p < 0.001 versus CTRL

Increased APC senescence is key in the reduced subcutaneous adipogenesis reported in hypertrophic obesity (Gustafson et al., 2019). Non‐obese FDR individuals also feature SAT hypertrophy due to impaired differentiation of resident APC (Arner et al., 2011). Thus, we examined the relationship between APC senescence and adipocyte hypertrophy—as an in vivo marker of adipogenic impairment (Henninger et al., 2014)—in the FDR and CTRL subjects of our study cohort. Interestingly, we found that increased percentage of SA‐β‐gal‐positive APC showed a strongly positive correlation with increased subcutaneous adipocyte size (Figure S12), indicating that senescence contributes to the inability of APC to undergo differentiation in the FDR subjects as well. Based on this finding, we investigated whether the *ZMAT3*‐induced senescence impairs adipogenesis in APC. Thus, *ZMAT3*‐overexpressing APC were subjected to adipogenic differentiation. As shown in Figure 4a,b, after differentiation, these cells accumulated significantly less lipids than EV‐transfected APC, demonstrating that the overexpression of *ZMAT3* in APC causes senescence and is accompanied by inhibition of adipogenesis. We also examined the ZMAT3 expression in both FDR and CTRL APC in relation to the ability of these progenitor cells to undergo adipocyte differentiation. As presented in Figure 4c,d, the ZMAT3 protein was increased in poorly differentiated FDR APC while remaining unchanged in normally differentiated CTRL APC. Thus, the expression of ZMAT3 needs to be tightly controlled in APC to preserve their ability to undergo normal adipocyte differentiation. Together, these data suggested that increased ZMAT3 expression in APC contributes to reduced subcutaneous adipogenesis in FDR subjects by a senescence‐dependent mechanism.

### 2.6 Effects of senolytics on *ZMAT3* DNA methylation profile and adipocyte differentiation in FDR APC

**FIGURE 5:**
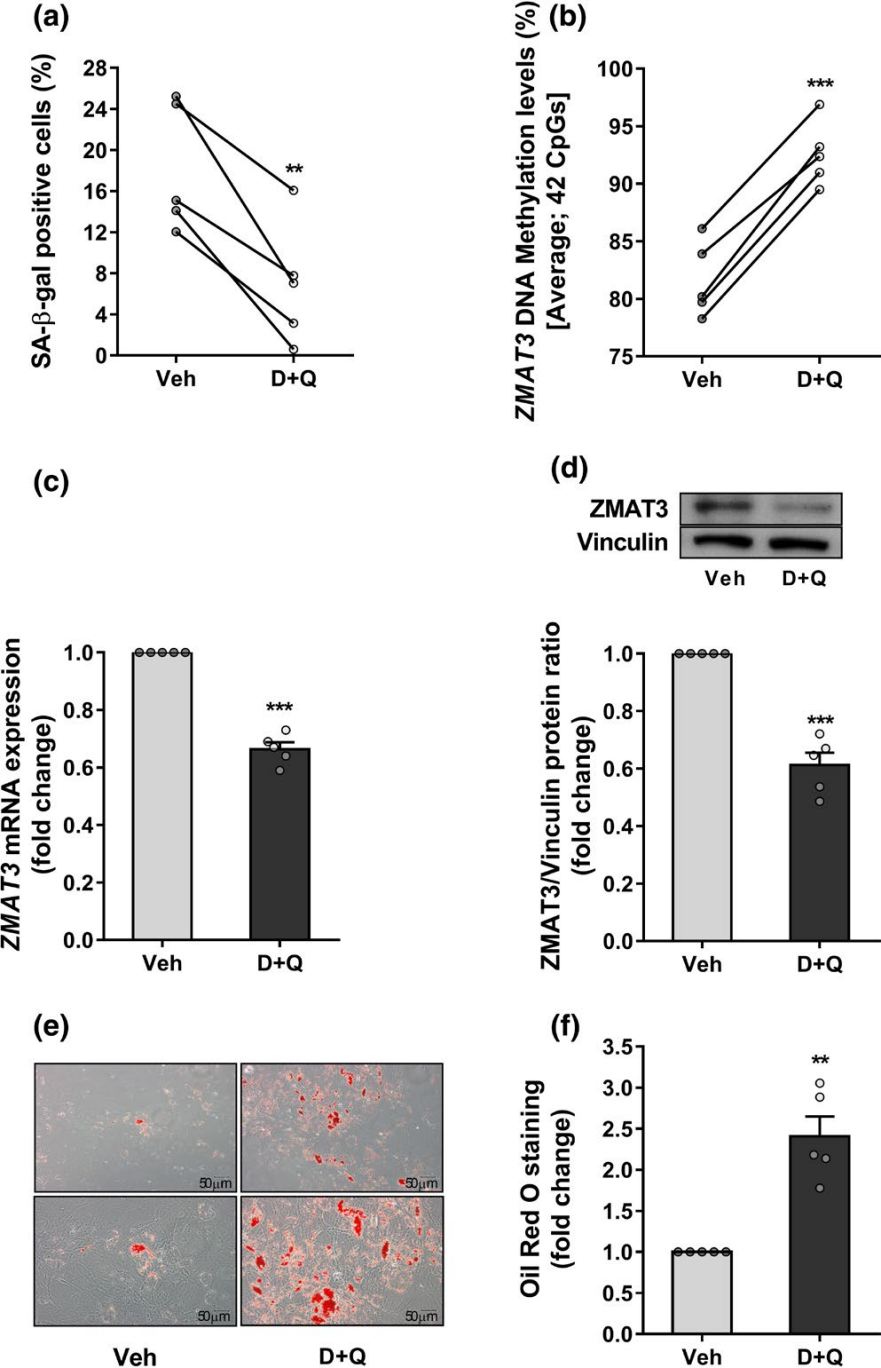
Effects of senolytics on *ZMAT3* DNA methylation and adipocyte differentiation in FDR APC. Five biologically independent APC samples randomly selected in the FDR group were treated with D+Q or vehicle (Veh) for 72 h. (a) Flow cytometric detection of the SA‐β‐gal‐positive cells in FDR APC treated with D + Q or Veh. Values are presented as percentage (%). (b) Changes in average DNA methylation levels at 42 CpGs within the *ZMAT3* DMR were detected by BS in D+Q‐treated FDR APC compared to Veh‐treated FDR APC. (c) The fold change of the *ZMAT3* mRNA was assessed by qPCR. Expression was normalized first to *RPL13A* and then to expression in Veh‐treated FDR APC. (d) The fold change of the ZMAT3 protein was measured by Western blot in D + Q‐ versus Veh‐treated FDR APC. Vinculin served as a loading control. The upper figure shows representative blots; the lower figure shows result quantitation. (e,f) After either D+Q or Veh treatment, the FDR APC were differentiated for 15 days. Oil Red O staining was used to assess the degree of differentiation. (e) Representative microphotographs showing lipid accumulation of FDR APC treated with D+Q (right) or Veh (left) at diff. day 15. Upper panel at 10x magnification; bottom panel at 20x magnification. Scale bar 50 μm. (f) Bar graph shows photometric quantification of Oil Red O staining measured at 490 nm. Results are normalized to the absorbance in Veh‐treated FDR APC. (a,b) Data are shown as scatterplot with lines joining paired points. (c,d,f) Data are shown as mean ±SEM. Dots represent individual level data. (a‐d,f) Significance was determined by paired Student's *t*‐test. **p < 0.01, ***p < 0.001 versus Veh

To search for additional evidence supporting the involvement of *ZMAT3* epigenetic dysregulation in senescence induction and the consequent adipogenic impairment in APC of FDR subjects, we tested the effects of senolytic combination Dasatinib plus Quercetin (D+Q) on these progenitor cells. D+Q treatment was adopted as this combination has been previously reported to efficiently clear senescent APC from mouse and human cell cultures, from old as well as insulin‐resistant mice, AT from obese diabetics and individuals with diabetic kidney disease nevertheless preserving viability of cycling‐competent cells (Kirkland & Tchkonia, 2020; Zhu et al., 2015). As shown in Figure 5a, treatment of APC from FDR subjects with D+Q for 3 days reduced the percentage SA‐β‐gal‐positive cells by 60%. This reduction was also paralleled by downregulation of both the P53 senescence marker and its target *CDKN1A* gene (Figure S13a,b). We further observed that the SASP factors IL6, MCP1, RANTES, IL8, and MIP1b were lower in the media conditioned by FDR APC treated with D+Q as compared to the media conditioned by vehicle‐treated FDR APC (Table S2). Importantly, the removal of senescent cells after D+Q treatment restored higher DNA methylation levels at the *ZMAT3* intronic DMR in FDR APC (Figure 5b). *ZMAT3* expression declined both at mRNA and protein levels in FDR APC upon D+Q treatment (Figure 5c,d). Thus, in these subjects, hypomethylation of the *ZMAT3* intronic DMR seemed to represent an epigenetic signature of senescent APC, while senolytic clearance of the senescent APC abrogated this aberrant signature.

Finally, we evaluated whether FDR individuals benefit from senotherapy. To this end, we examined the ability of FDR APC to undergo adipocyte differentiation after D+Q treatment. Gene expression of the key adipogenic factor *peroxisome proliferator*‐*activated receptor gamma 2* (*PPARG_2_*) was increased in the D+Q‐treated FDR APC *versus* vehicle‐treated FDR APC (Figure S13c). Since *PPARG_2_* is necessary for adipogenesis (Rosen et al., 2000), these data revealed that senolytic treatment improves adipogenic potential of FDR APC. To corroborate this evidence, D+Q‐ and vehicle‐treated FDR APC were then subjected to adipocyte differentiation. As shown in Figure 5e,f, D + Q treatment was capable to ameliorate adipogenic capacity of FDR APC. Taken together, these data confirmed the direct contribution of senescent APC to restricted adipogenesis in FDR and proved that functional impairment of FDR APC can be pharmacologically reverted.

### 2.7 Age‐, senescence‐, and T2D‐associated *ZMAT3* expression in SAT

**FIGURE 6:**
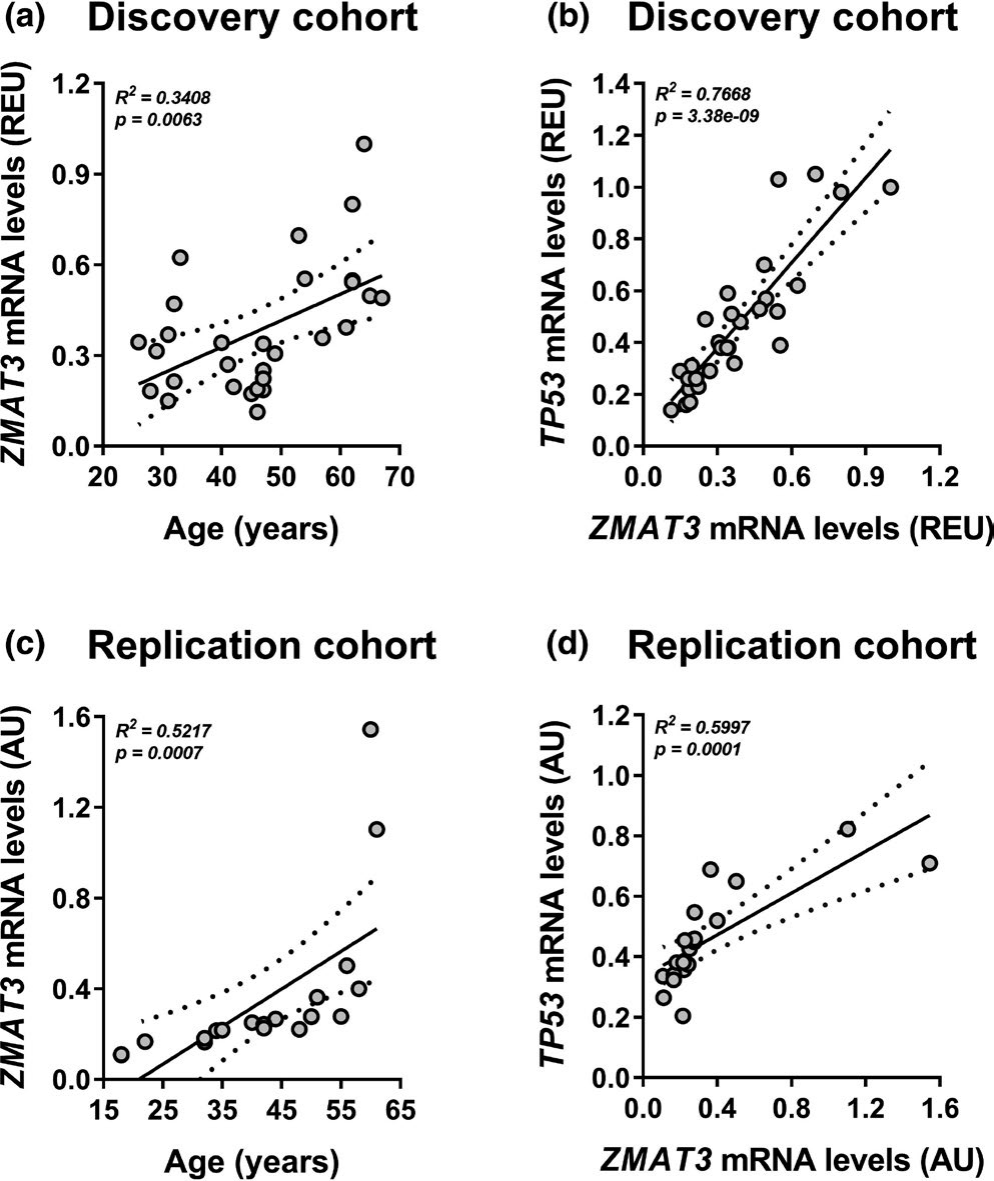
Age and senescence association with *ZMAT3* expression in SAT. (a,b) *ZMAT3* and *TP53* mRNA levels were measured by qPCR in subcutaneous adipose cells from 29 individuals aged 26 to 67. Values are presented as relative expression units (REU). (a) Scatter plot shows the association between the age of study participants and *ZMAT3* mRNA levels (*regression coefficient* = 0.0080, adjusted for BMI). (b) Scatter plot depicts the association between *ZMAT3* and *TP53* mRNA levels in subcutaneous adipose cells (*regression coefficient* = 1.1004, adjusted for BMI). (c,d) *ZMAT3* and *TP53* mRNA levels were measured by qPCR in SAT from 20 women aged 18 to 61. Values are presented as absolute units (AU). (c) Scatter plot shows the association between the age of study participants and *ZMAT3* mRNA levels (*regression coefficient* = 0.0186, adjusted for BMI). (d) Scatter plot depicts the association between *ZMAT3* and *TP53* mRNA levels in SAT (*regression coefficient* = 0.3558, adjusted for BMI). (a‐d) The association analyses were adjusted for BMI by using multiple linear regression models. R^2^ and *p* values for the whole models are shown in the graphs. Dashed lines indicate the 95% confidence intervals for the regression line

Increased senescent cell burden in SAT contributes to the development of T2D in elderly (Spinelli et al., 2020). Accordingly, we have investigated whether, *in vivo*, the upregulation of *ZMAT3* transcription, previously identified as a major age‐related gene in most tissues including SAT (Dong et al., 2021), associates with human aging and T2D. To this end, we measured mRNA levels of *ZMAT3* in subcutaneous adipose cells from individuals aged 26 to 67. The clinical characteristics of these subjects have been previously reported (Gustafson et al., 2019). The subjects were then stratified according to *ZMAT3* expression levels, and two subgroups expressing *ZMAT3* levels above (*n *= 14) or below (*n *= 15) *ZMAT3* median values were identified. As shown in Table S3, the higher *ZMAT3* expressors also featured higher *TP53* mRNA levels, were significantly older, had significantly greater T2D prevalence, and showed higher BMI. Importantly, multiple regression analysis further revealed that age was significantly associated with *ZMAT3* gene expression in this group, similar to *TP53* expression (Figure 6a,b). To validate the association between *ZMAT3* expression and age, we sought to replicate the age‐related upregulation of *ZMAT3* in the SAT from an independent cohort of healthy women with similar age range as those in the previously analyzed cohort (D'Esposito et al., 2012). Accordingly, these subjects were divided, based on *ZMAT3* mRNA levels, in two groups, low (*n *= 10) and high (*n *= 10) expressors, defined, respectively, by expression levels below or above the median value. As previously shown, the higher *ZMAT3* expressors were significantly older and had higher *TP53* mRNA levels (Table S4). Nevertheless, age was positively associated with *ZMAT3* mRNA in this group, as was *TP53* expression (Figure 6c,d). In addition, we examined the ZMAT3 protein in all of the available SAT samples in the replication cohort and compared the high versus low *ZMAT3* expressors. Once again, this comparison revealed that protein analysis of ZMAT3 largely reproduced the transcriptional data (Figure S14a). Furthermore, a positive correlation between *ZMAT3* mRNA and protein measurements across the same SAT samples was observed (Figure S14b). Taken together, these findings support that the age‐related *ZMAT3* upregulation may have functional consequences in SAT through senescence induction, thereby impacting T2D risk. Finally, to further validate the link between *ZMAT3* upregulation and T2D, we interrogated the GEO public functional genomics data repository and analyzed data from the GSE27951 data set which contains the transcriptional signature of SAT from subjects with normal glucose tolerance and T2D. Our analysis revealed that *ZMAT3* expression was significantly higher (*p *< *0*.*05*) in the T2D patients in this data set (data not shown). Taken together, our findings and the *in*‐*silico* data indicated that, in human SAT, an increase in *ZMAT3* transcription is a feature of the elderly and T2D subjects.

## 3 DISCUSSION

Capability to activate mechanisms of SAT hyperplasia in response to obesity and aging is critical for metabolic health as development of T2D in obesity and/or in the elderly can be, in part, prevented as long as SAT expansion is driven by recruitment of new adipocytes rather than by hypertrophy of the pre‐existing cells (Gao et al., 2020; Smith & Kahn, 2016). Accordingly, it has been proposed that, by impairing *de novo* adipogenesis, APC senescence contributes to the increased risk of T2D in both aged and obese subjects (Wissler Gerdes et al., 2020). Despite this evidence, the molecular mechanisms leading to APC senescence and their impact on T2D risk remain unclear. SAT from the FDR individuals investigated in the present work featured both decreased adipogenesis and adipocyte hypertrophy which is a trait of FDR and best seen when not obese (Smith & Kahn, 2016). Even though these subjects were young and non‐obese, we hypothesized that these SAT changes were caused by early senescence of their APC. In addition, we have recently demonstrated that the methylome of SAT‐derived APC from these same FDR is characterized by extensive hypomethylation (Parrillo et al., 2020) as also demonstrated in other SNC (Cruickshanks et al., 2013). Thus, we investigated whether the DNA hypomethylation associated with T2D familiarity causes early senescence in the APC of subjects who are FDR, thereby contributing to their high risk to develop T2D.

SNC are characterized by irreversible growth arrest and a flat vacuolated morphology (Herranz & Gil, 2018). They secrete bioactive SASPs which determine the fate of the surrounding cells and *milieu* (Song et al., 2020). The most reliable approach to SNC identification involves the use of a panel of different markers, including SA‐β‐gal, loss of *LMNB1*, increased levels of both cell cycle inhibitors (*e*.*g*., *CDKN1A*), and commonly secreted SASPs (Hernandez‐Segura et al., 2017; Wiley et al., 2017). Here, we show that SNC burden, as assessed by analyzing all these marks, is significantly increased in the APC from subjects who are FDR. These cells feature enhanced secretion of the proinflammatory SASP factors IL6, MCP1, RANTES, IL8, and MIP1b (Freund et al., 2010). Secretion of these molecules in the conditioned media of the APC from the individuals who were FDR was significantly reduced following senolytic clearance of SNC, thereby proving that these senescent APC have acquired a proinflammatory SASP. Previous studies demonstrated that FDR subjects display SAT and systemic inflammation (Henninger et al., 2014; Ruotsalainen et al., 2006). At least in part, this inflammatory profile is likely due to APC secretion of the same SASP factors that have been shown to cause defective adipogenesis, inflammation, aberrant adipocytokine production, and IR in both elderly and obese individuals (Liu et al., 2020; Tchkonia et al., 2020). Thus, senescent APC appear to represent a source of multiple factors contributing to the SAT dysfunction and metabolic abnormalities associated with T2D familiarity in FDR subjects.

Epigenetic modifications, including loss of DNA methylation, are key in regulating the senescence phenotype (Atkinson & Keith, 2007; Cheng et al., 2017). Interestingly, major risk factors for T2D (aging, obesity, T2D familiarity), which are associated with increased APC senescence, contribute to IR by affecting the AT methylome in non‐diabetic subjects (Davegårdh et al., 2018; Parrillo et al., 2019). Furthermore, lifestyle interventions (diet, exercise, weight loss) that prevent T2D development by inducing DNA methylation changes in the AT, further exert their protective effects by preventing APC senescence (Justice et al., 2018; Most et al., 2017). Therefore, in this work, it has been of interest to establish how DNA methylation contributes to T2D risk by promoting APC senescence. Our previous methylome findings in the APC of subjects who are FDR showed that most of genomic regions differentially methylated in these individuals were less methylated and many overlapped with senescence‐related genes (Gorgoulis et al., 2019; Parrillo et al., 2020). These genes include *ZMAT3*, which belongs to the p53‐dependent growth‐inhibiting and tumor suppressor pathways (Hellborg et al., 2001; Janic et al., 2018). The *ZMAT3* gene encodes a zinc‐finger RNA‐binding protein, highly conserved from fish to human, which is involved in post‐transcriptional regulation of gene expression by affecting mRNA stability and translation (Hellborg et al., 2001). A recent study also uncovered a novel function for ZMAT3 in RNA homeostasis by modulating alternative splicing, resulting in multifaceted effects on several cellular processes (Bieging‐Rolett et al., 2020). *ZMAT3* upregulation has been reported in both human SNC and aged tissues (Chaturvedi et al., 2015; Lee et al., 2014; Marthandan et al., 2016; Yang et al., 2016). In a recent analysis of 17,382 whole transcriptome profiles in 54 tissue types from 979 human donors aged 20 to 79 and included in the GTEx database (V.8), *ZMAT3* emerged as one of the top ten age‐related and differentially expressed genes. Importantly, *ZMAT3* also showed a significant positive association with age in multiple tissues, including SAT (Dong et al., 2021). In addition, *ZMAT3* hypomethylation has been found in the cerebellum of subjects with Down Syndrome, which has been described as a human condition of accelerated aging (Gensous et al., 2019; Mendioroz et al., 2016). However, whether and how *ZMAT3* induces APC senescence has been revealed in this work for the first time. In the present report, we have shown that, in APC from FDR, the reduced methylation occurring at the *ZMAT3* DMR is accompanied by increased *ZMAT3* expression. Also, we demonstrated that hypomethylation at this gene region directly increased transcriptional activity of *ZMAT3 in vitro*, which may occur *in vivo* as well. Indeed, the *ZMAT3* DMR overlaps with the intronic *chr3*:*179032651*–*179039599* region of the *ZMAT3* gene annotated as regulatory feature based on the Ensembl regulatory build (Zerbino et al., 2015). Although there is a significant difference in *ZMAT3* DNA methylation between FDR and CTRL subjects, the percentage changes are small, with an 8% mean DNA methylation difference between groups. This result, however, is consistent with previous findings that environmental factors linked to metabolic diseases such as T2D alter DNA methylation at specific genes in a subtle manner (Kirchner et al., 2016). Nevertheless, we confirmed that FDR‐associated DNA methylation changes at the *ZMAT3* DMR have a functional impact on its expression. In addition, a recent meta‐analysis of age‐annotated methylation data available from GEO database has shown that DNA methylation changes at age‐predictive loci, which are enriched in intergenic regions and gene enhancers, are small in magnitude (<5%) across the lifespan (Porter et al., 2021). This could be due to an increase in SNC, a relatively rare cell subtype in all tissues (Porter et al., 2021), which may also occur in FDR. These new findings prompted us to hypothesize that the methylation changes causing *ZMAT3* upregulation also contribute to the early APC senescence occurring in FDR. Consistent with this hypothesis, we found that methylation levels at the *ZMAT3* DMR negatively correlated with appearance of the senescence phenotype in the APC, while *ZMAT3* mRNA and protein levels positively correlated. In addition, we have shown that the premature senescence determined by APC exposure to the demethylating agent 5‐AZA was accompanied by reduced DNA methylation at the *ZMAT3* DMR and increased expression of the gene. The pro‐senescence effect of 5‐AZA treatment was prevented by siRNA‐mediated silencing of *ZMAT3*, indicating that hypomethylation‐induced senescence is, at least partly, attributable to *ZMAT3* upregulation. Consistently, reduction of senescent cell burden by D+Q in the FDR APC resulted in a significant reestablishment of higher DNA methylation levels at the same intronic region, with concomitant decrease in *ZMAT3* expression. Thus, epigenetic dysregulation of *ZMAT3* is a bona fide marker of APC senescence. This conclusion is further strengthened by the evidence that *ZMAT3*‐overexpressing APC mirror the senescence phenotype observed in FDR APC based upon SA‐β‐gal expression, *CDKN1A* upregulation, loss of *LMNB1* and SASP acquisition. The SASP profile acquired by the *ZMAT3*‐overexpressing APC does not completely replicate that seen in the APC from FDR. Nevertheless, increased secretion of both RANTES and IL8 was detected in media conditioned by APC where senescence was induced by *ZMAT3*. Other factors, along with *ZMAT3*, likely contribute to determining inflammatory‐type SASP in the APC of FDR.

ZMAT3 regulates a wide range of transcripts which are implicated in several biological processes, including cell cycle, immune system function, and metabolic responses (Bersani et al., 2014, 2016; Vilborg et al., 2009). It is also a positive regulator of p53, which is critical for senescence induction. ZMAT3 maintains high levels of P53 by blocking deadenylation of its mRNA (Vilborg et al., 2009). In addition, *Bieging*‐*Rolett* et al. have recently demonstrated that ZMAT3 promotes full P53 activity by triggering mRNA decay of its MDM4 and MDM2 inhibitors (Bieging‐Rolett et al., 2020). These reports have highlighted the growth‐suppressive role of ZMAT3 in human cells, particularly when P53 function is intact, suggesting that ZMAT3 is most active when its signaling occurs through the p53‐dependent pathways (Bieging‐Rolett et al., 2020). Consistently, we have found that P53 levels significantly increased in both *ZMAT3*‐overexpressing APC and FDR APC. Since *ZMAT3* is also a direct transcriptional target of P53 (Vilborg et al., 2009), the induction of P53 by ZMAT3 in APC may initiate a positive feedback loop to counterbalance MDM2‐ and MDM4‐dependent P53 inhibition and increase P53 activity. While the effect of ZMAT3 on the P53 inhibitors MDM4 and MDM2 has not been explored in this study, the findings that the expression of the p53 target gene *CDKN1A* significantly increased in both *ZMAT3*‐overexpressing APC and FDR APC support the role of ZMAT3 in increasing P53 activity. This conclusion is further corroborated by the evidence that ZMAT3 specifically regulates *p21* expression at the transcriptional level by increasing the capability of P53 to bind the *CDKN1A* promoter *in vitro*, which may occur *in vivo* as well. Thus, upregulation of *ZMAT3* in the APC triggers the induction of P53, which in turn leads to the transcription of the p21‐encoding *CDKN1A* gene. Consistently, we further show that pharmacological inhibition of P53 transcriptional activity in *ZMAT3*‐overexpressing APC significantly reduces *CDKN1A* mRNA levels, confirming that *ZMAT3*‐induced *CDKN1A* expression is p53‐dependent. Interestingly, P53 inhibition by PFTα in *ZMAT3*‐overexpressing APC also prevents cell senescence. Based on these findings, we conclude that *ZMAT3* causes APC senescence by activating the p53/p21 pathway. Cell senescence is a potent anticancer mechanism, and the p53/p21 pathway functions as tumor suppressor (Campisi, 2005). Accordingly, ZMAT3 has been identified as a tumor suppressor in lung and liver cancers, as well as human carcinomas (Bieging‐Rolett et al., 2020). Thus, our findings also suggest that the ZMAT3‐positive feedback to P53 may account for some ZMAT3 tumor suppressor activity.

Previous investigators showed that, in cancer cell lines, ZMAT3 induced *p21* mRNA decay with no change in p53 and suggested that these events cause premature senescence upon *ZMAT3* depletion (Kim et al., 2012). However and consistent with our findings, other investigators also showed that *ZMAT3* and *p21* expression is induced with similar kinetics in both normal and cancer cells (Hellborg et al., 2001; Muys et al., 2020; Parikh et al., 2014), indicating that the effects of *ZMAT3* may depend on the cellular context. Based on the evidence presented in this work, we propose that the epigenetic dysregulation of *ZMAT3* induces premature senescence in APC of FDR by activating the p53/p21 pathway. Our results are consistent with the seminal study by *Minamino* et al. who demonstrated the key role of the p53/p21 pathway in the induction of premature senescence in the AT in both of obese and insulin‐resistant mice and in that of T2D patients. These authors proved that adipocyte‐specific ablation of p53 reduced AT senescence and inflammation and improved IR in diabetic mice. Conversely, p53 overexpression caused AT senescence along with a proinflammatory environment that impaired insulin sensitivity (Minamino et al., 2009). Thus, the *ZMAT3*‐induced activation of the p53/p21 pathway links premature APC senescence and accelerated development of IR in subjects who are FDR. These observations led us to investigate whether *ZMAT3* upregulation occurs *in vivo* and whether it associates with aging and T2D. Interestingly, analysis of SAT from elderly and T2D individuals revealed increased *ZMAT3* expression. Furthermore, in these subjects, the age‐related upregulation of the *ZMAT3* gene was strongly associated with elevated expression of the *P53* senescence marker. Thus, *ZMAT3* upregulation occurs in both FDR and in elderly subjects, contributing to SAT dysfunction and T2D risk.

In the APC, senescence is a major negative regulator of adipogenesis (Spinelli et al., 2020). Consistently, in this work, we show that *ZMAT3* overexpression in APC induces senescence accompanied by impaired adipogenesis. Also, ZMAT3 levels are enhanced in poorly differentiated APC of FDR subjects, as previously shown for its target P53 (Gustafson et al., 2019). P53 activation is known to inhibit adipocyte differentiation. Indeed, P53 needs to be downregulated before APC can differentiate into insulin‐responsive adipocytes (Lee et al., 2020). Thus, in APC from subjects who are FDR, the upregulation of *ZMAT3* caused by hypomethylation appears to maintain P53 upregulated, induce senescence, and increase T2D risk by restraining adipogenesis.

In the present work, we have provided the first evidence that FDR might benefit from senolytic therapy. Indeed, the combination of D+Q has recently emerged as an attractive therapeutic strategy by allowing selective clearance of senescent APC both *in vitro* and *in vivo* (Robbins et al., 2020). Indeed, intermittent oral administration of D+Q improves insulin sensitivity in obese mice by restoring the ability of APC to differentiate into insulin‐responsive adipocytes and by reducing AT hypertrophy and inflammation (Palmer et al., 2019). Furthermore, in patients with T2D complicated by renal dysfunction, a 3‐day oral administration of D + Q is effective in reducing SNC burden and macrophage infiltration in abdominal SAT and in reducing plasma levels of the main SASP mediators (Hickson et al., 2020). In this study, we have demonstrated that the expression of senescence markers and SASP factors decreased in the FDR APC after 3‐day treatment with D+Q. In agreement with the senolytic activity on senescent APC and the selective survival of non‐senescent APC in the culture, we found higher DNA methylation and decreased expression of *ZMAT3* in the D+Q‐treated *versus* vehicle‐treated FDR APC, indicating that the FDR‐associated *ZMAT3* DNA methylation signature marks senescent APC.

In SAT from subjects who are FDR, the downregulation of *PPARG_2_* impairs adipocyte differentiation leading to adipocyte hypertrophy and IR (Acosta et al., 2016; Yang et al., 2004). Interestingly, we found that cultured APC from FDR exhibit higher *PPARG_2_* expression and improved adipogenic capacity upon senescent cell clearance following D+Q treatment. Consistent with an effect mediated by clearance of senescent APC, D+Q action on adipogenesis persisted after removal of these agents from the culture medium. The possibility to introduce senolytics to improve insulin sensitivity in humans needs to be further investigated in future studies. Lack of biomarkers that unambiguously discriminate senescent and non‐senescent cells is one difficulty in evaluating senolytic therapies in clinical trials. We suggest that DNA methylation profiling of *ZMAT3* may be used for this purpose.

In conclusion, our findings identify early APC senescence as a key player in the SAT dysfunction occurring in healthy individuals who are FDR of T2D patients and reveal a previously unknown role of *ZMAT3* hypomethylation in determining these events. In addition, we provide further evidence that senolytic‐induced clearance of SNC improves APC adipogenesis and might contribute to decreasing diabetes risk in these FDR.

## 4 EXPERIMENTAL PROCEDURES

A detailed Methods section is available in Appendix S1.

### 4.1 Study participants

Twenty‐four subjects were selected from the EUGENE2 consortium (Laakso et al., 2008). A 50:50 distribution of participants' gender was achieved; mean age was 40.6 years (SEM:1.6 years); mean BMI was 24.9 Kg/m^2^ (SEM:0.4 Kg/m^2^). The clinical characteristics of these individuals have been previously described (Laakso et al., 2008) and are presented in Table 2. These subjects were healthy, non‐obese and either had (*n *= 12; FDR) or did not have (*n *= 12; CTRL) one first‐degree relative with T2D. FDR subjects exhibited a significantly reduced insulin sensitivity and larger subcutaneous adipocytes when compared to CTRL subjects. There was no significant difference between two groups in age, sex, BMI, and body fat percent. All enrolled subjects, who provided written informed consent, underwent an abdominal SAT sampling from the paraumbilical region. The study protocol was approved by the Ethical Committee of the University of Gothenburg (ethical approval numbers S655‐03 and T492‐17) according to the Declaration of Helsinki.

**TABLE 2:** Clinical characteristics of FDR and CTRL subjects

Phenotypes	FDR subjects	CTRL subjects	*p* value
N (female/male)	12 (6/6)	12 (6/6)	>0.9999
Age, years	45.0 (35.0; 49.7)	38.0 (35.0; 46.0)	0.1386
BMI, Kg/m^2^	25.0 (23.9; 26.6)	24.9 (22.8; 26.7)	0.5800
Fat percent, %	27.7 (19.8; 31.4)	23.2 (20.1; 31.2)	0.5516
Waist to Hip Ratio (WHR)	0.92 (0.87; 0.96)	0.82 (0.74; 0.86)	*0.0021*
Subcutaneous adipocyte size, µm	101.0 (99.6; 104.6)	90.4 (87.0; 92.0)	*<0.0001*
f‐insulin, pmol/L	53.4 (49.3; 66.2)	31.9 (23.6; 44.4)	*0.0091*
fb‐glucose, mmol/L	4.7 (4.5; 5.2)	4.3 (4.1; 4.6)	*0.0070*
OGTT p‐glucose 2 h, mmol/L	6.5 (3.4; 8.1)	4.6 (4.0; 6.1)	*0.0346*

Note

Gender (female/male) is expressed as number. Other data are shown as median (first quartile‐Q1; third quartile‐Q3). Statistical differences between the two groups were tested using Mann–Whitney test (continuous variables) or Fisher's exact test (categorical variable). *p* value versus CTRL. *p *≤ 0.05 was considered statistically significant and it is italicised in the table.

Abbreviations: BMI, body mass index; CTRL, subjects with no diabetes familiarity; fb‐glucose, fasting blood glucose; FDR, first‐degree relatives of T2D subjects; f‐insulin, fasting insulin; OGTT, oral glucose tolerance test; p‐glucose, plasma glucose.

### 4.2 Isolation and culture of APC

APC were isolated from SAT samples and cultured as reported (Gustafson et al., 2019).

### 4.3 Adipogenic differentiation of APC

APC were differentiated into adipocytes as described (Gustafson et al., 2019). To examine lipid accumulation, differentiated APC were stained with Oil Red O as reported (Mirra et al., 2021).

### 4.4 Flow cytometry analysis

Flow cytometric forward scatter and side scatter density plots were applied to analyze APC size and structure as described (Ratushnyy et al., 2020). SA‐β‐gal activity was assessed as indicated (Debacq‐Chainiaux et al., 2009). Cell cycle analysis was performed as reported (Raciti et al., 2018).

### 4.5 Primer sequences

Sequences of all primers are shown in Table S5.

### 4.6 Bisulfite sequencing

Bisulfite sequencing was performed as reported (Desiderio et al., 2019; Raciti et al., 2017).

### 4.7 RNA isolation and qPCR

Total RNA was extracted using the AllPrep DNA/RNA Mini Kit. cDNA synthesis and qPCR were performed as described (Longo et al., 2016; Nigro et al., 2019).

### 4.8 Western blot

Protein extraction and immunoblotting were carried out as indicated (Pirone et al., 2019; Ungaro et al., 2012). Antibodies against ZMAT3 (ab191536, Abcam), P53 (sc‐126, Santa Cruz), and Vinculin (sc‐73614, Santa Cruz) were used for protein detection.

### 4.9 Statistical analysis

Data are presented according to proposed guidelines for basic science data visualization (Weissgerber et al., 2017). Biological replicates were collected from different samples, each isolated from different human specimens. The number of independent biological replicates (*n*) used in each experiment was indicated in the figure. Statistical analysis was performed with GraphPad Prism 6.0 software (GraphPad Software Inc) and R statistical platform. Normal distribution of continuous variables was tested using the Shapiro–Wilk test. Normally distributed data were compared between groups by unpaired Student's *t*‐test (two‐tailed). Within‐group comparisons between matched samples were performed using paired two‐tailed Student *t*‐test or one‐way repeated measures ANOVA followed by Tukey's multi‐comparison test, as appropriate. Not normally distributed data were compared between groups by Mann–Whitney test (two‐tailed). The correlation between quantitative variables was tested by Spearman's rank correlation test. The association between age or *TP53* mRNA levels and *ZMAT3* expression in both subcutaneous adipose cells and SAT was tested by multiple regression analysis adjusting for BMI. BMI was fixed as covariate to account for the potential confounding effect of the association between this variable and cellular senescence and mRNA expression in human SAT. *p *≤ 0.05 was considered statistically significant.

## CONFLICT OF INTEREST

The authors declare no competing interests.

## AUTHOR CONTRIBUTIONS

R.S. designed research, interpreted the data, and wrote the manuscript. P.F_L_. acquired, analyzed, and interpreted the data. L.P., F.Z., M.L., A.N., and B.G. acquired and analyzed the data. V.D. and A.D. acquired the data. P.F_O_., C.M., G.A.R., R.N., and U.S. critically reviewed the manuscript. F.B. conceived the study, critically reviewed the manuscript and edited it. All authors approved the final manuscript.

## Supporting information

Fig S1

Fig S2

Fig S3

Fig S4

Fig S5

Fig S6

Fig S7

Fig S8

Fig S9

Fig S10

Fig S11

Fig S12

Fig S13

Fig S14

Table S1

Table S2

Table S3

Table S4

Table S5

Appendix S1

## Data Availability

The data that support the findings of this study are available from the corresponding author upon reasonable request.
