# Supplementary figures and images for: ZMAT3 hypomethylation contributes to early senescence of preadipocytes from healthy first‐degree relatives of type 2 diabetics
Source: Aging Cell. 2022 Feb 11;21(3):e13557. doi: 10.1111/acel.13557 (PMC8920444; doi:10.1111/acel.13557)

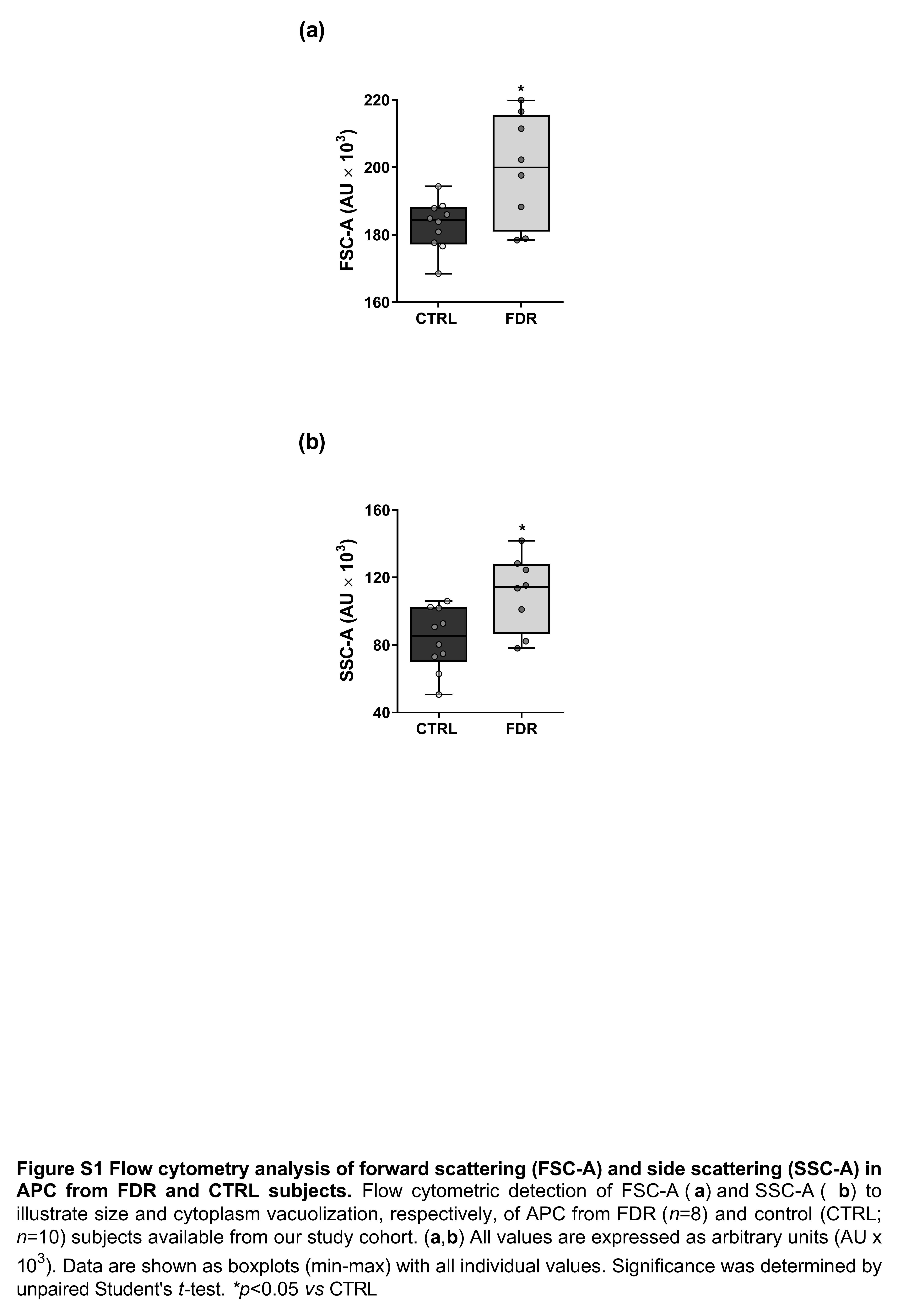

Supplement: Supplementary file 1 — Fig S1 [file ACEL-21-e13557-s017.jpg]

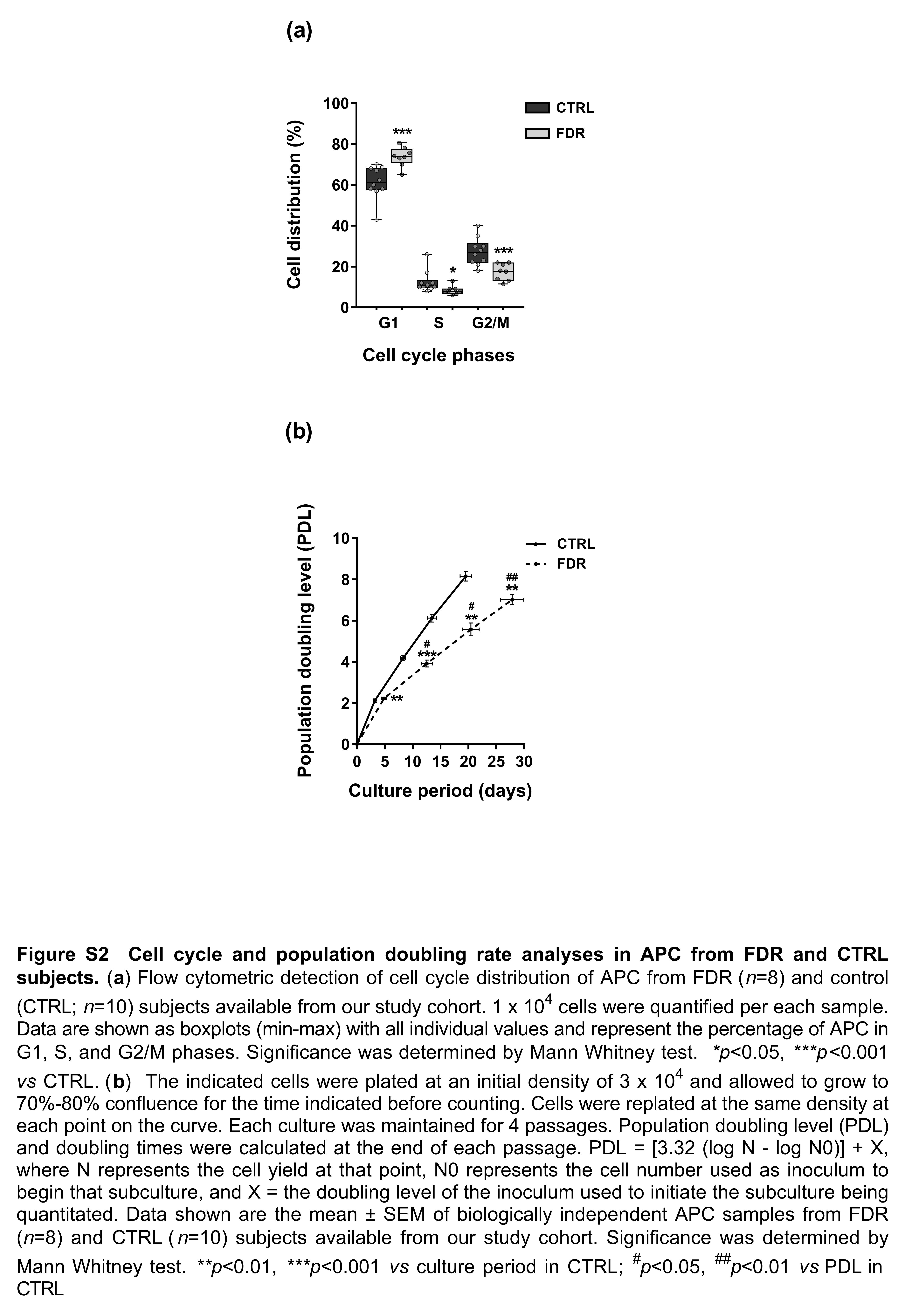

Supplement: Supplementary file 2 — Fig S2 [file ACEL-21-e13557-s013.jpg]

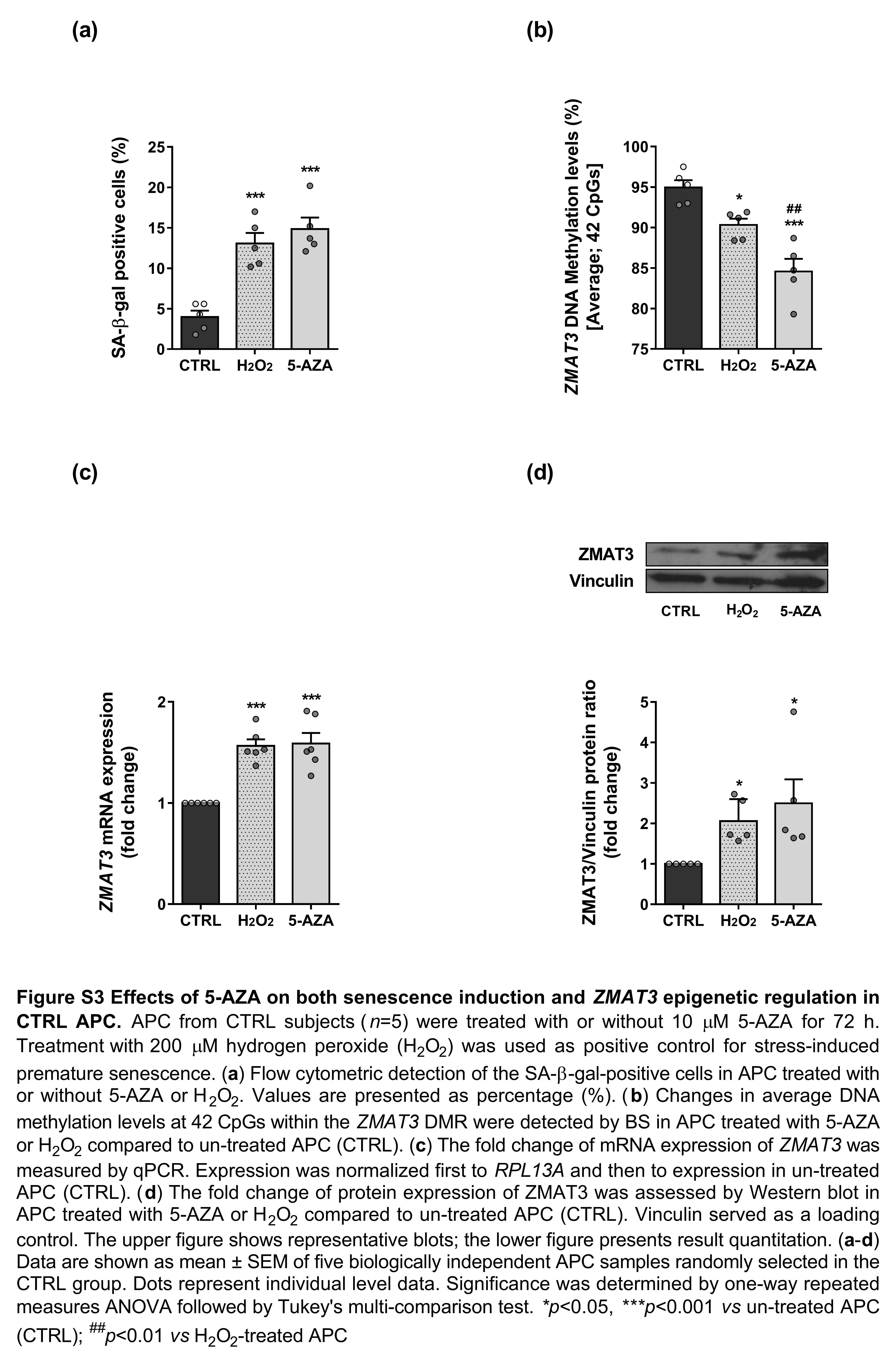

Supplement: Supplementary file 3 — Fig S3 [file ACEL-21-e13557-s014.jpg]

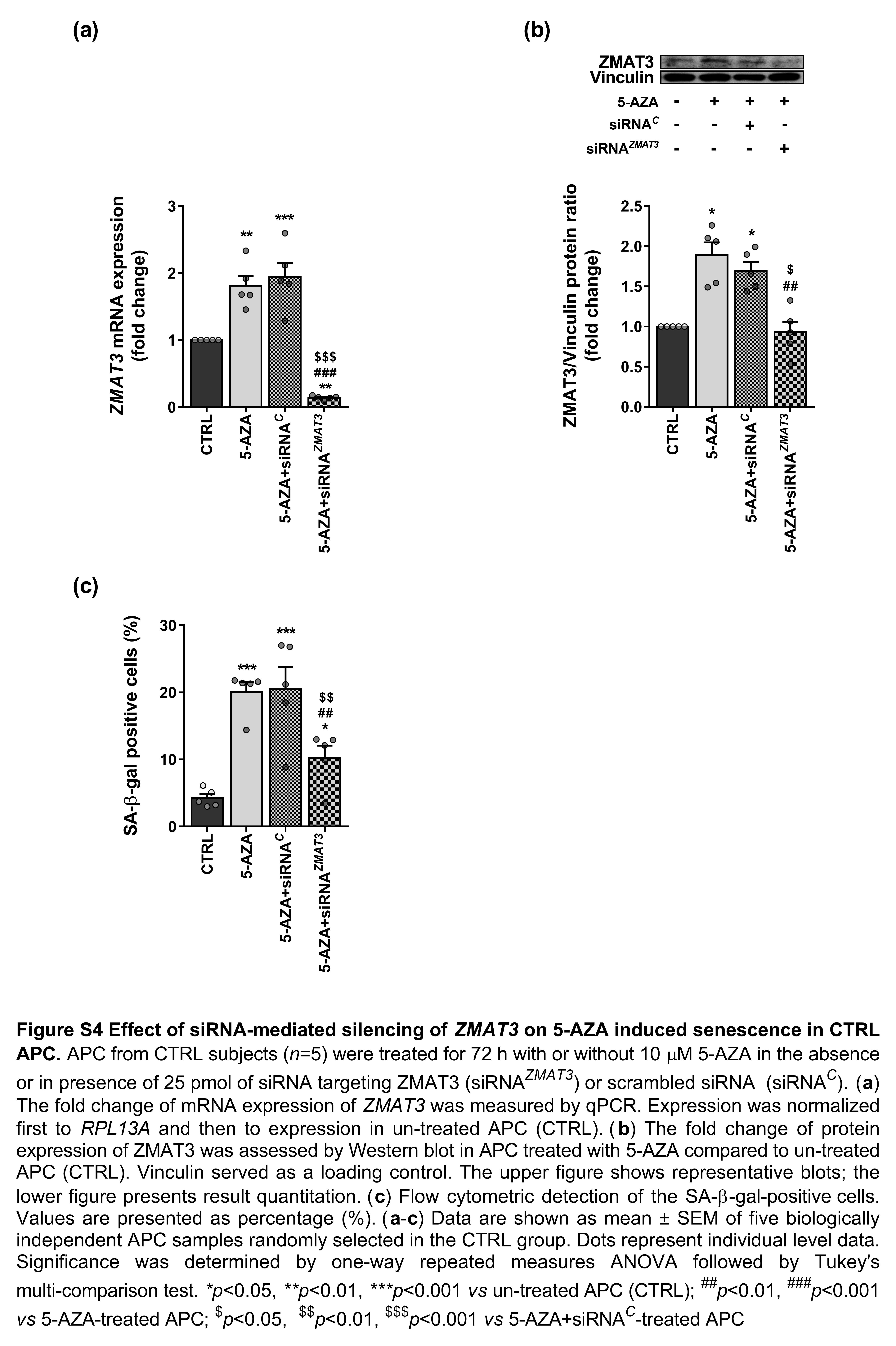

Supplement: Supplementary file 4 — Fig S4 [file ACEL-21-e13557-s005.jpg]

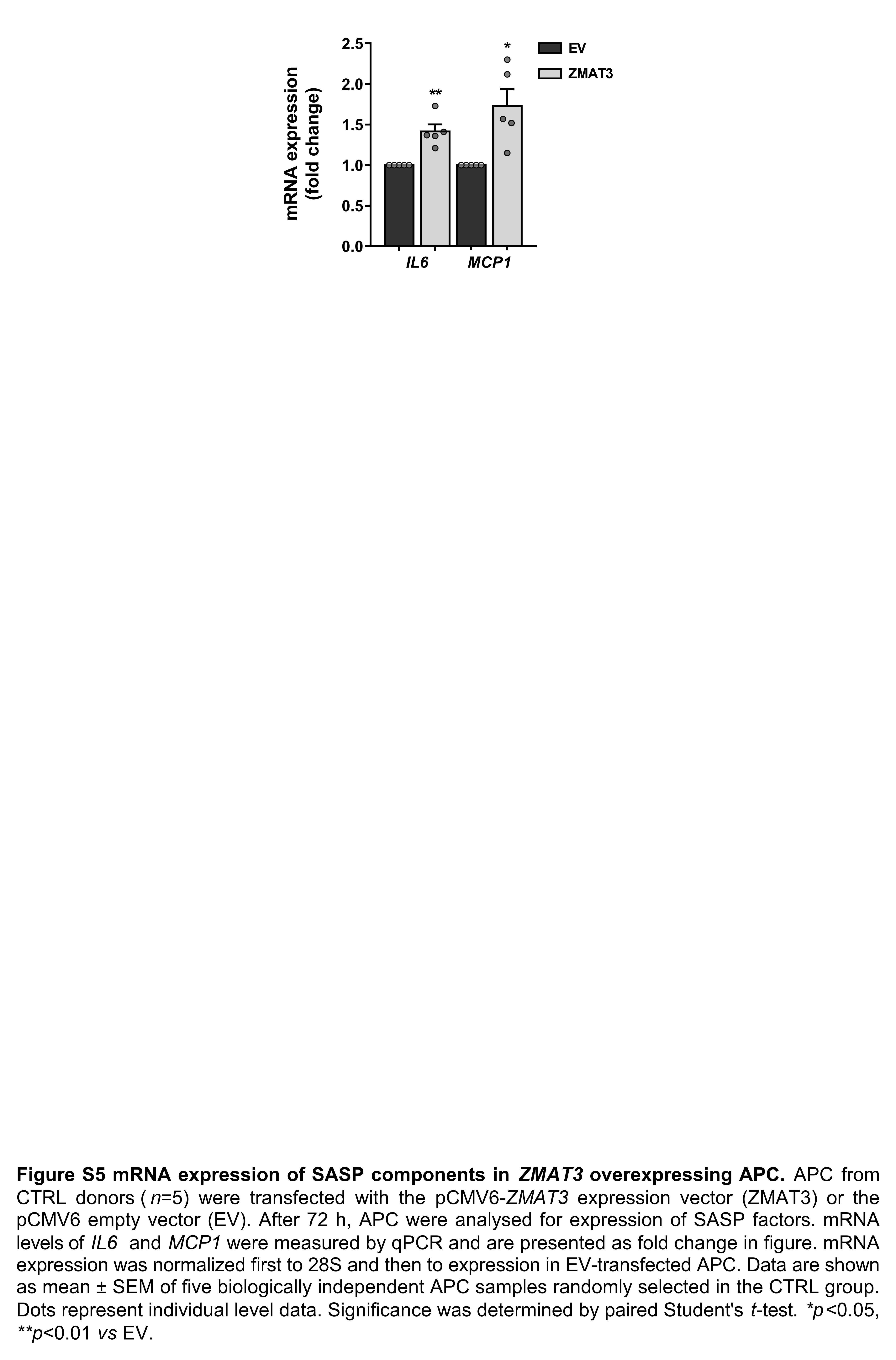

Supplement: Supplementary file 5 — Fig S5 [file ACEL-21-e13557-s003.jpg]

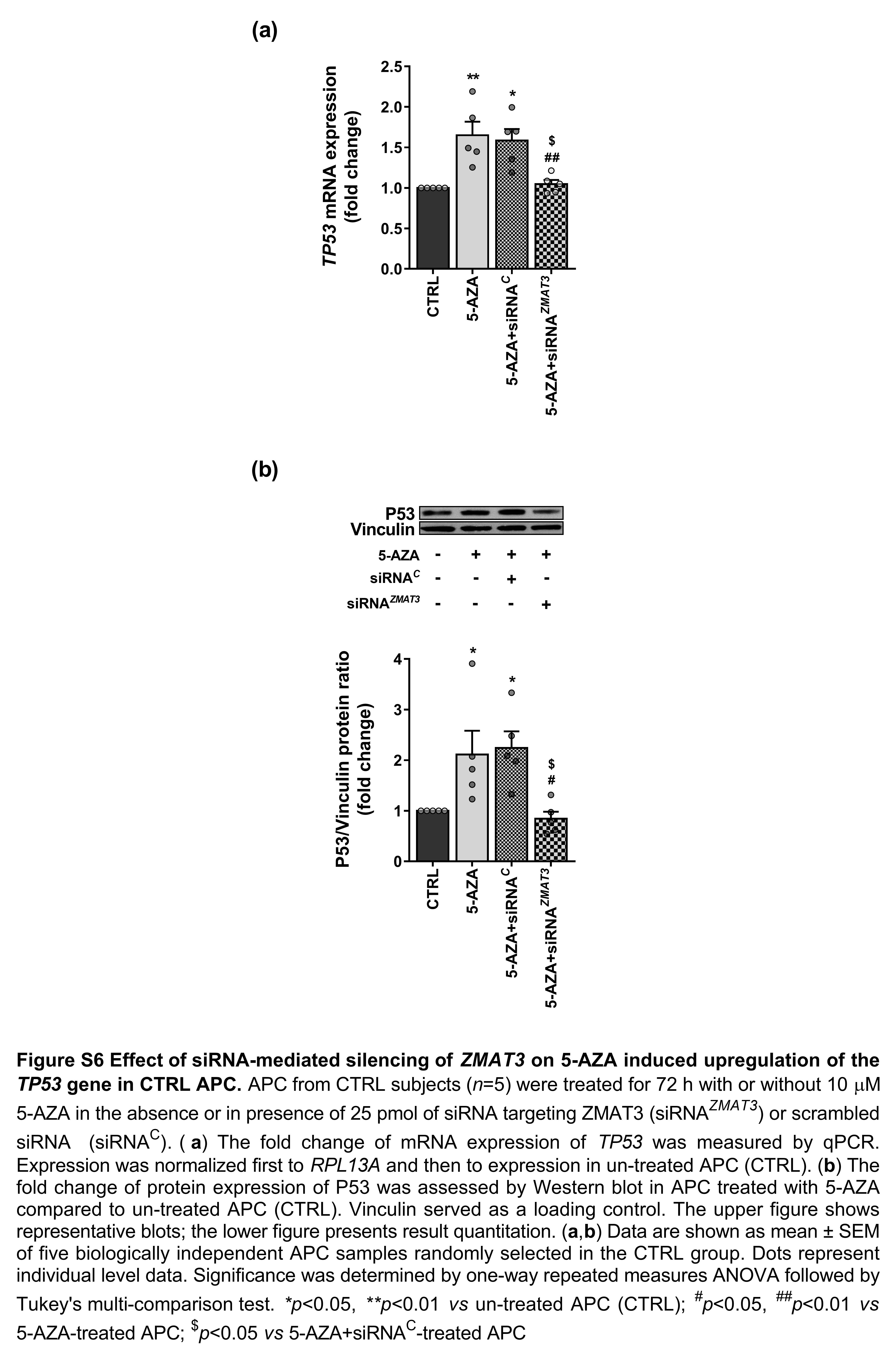

Supplement: Supplementary file 6 — Fig S6 [file ACEL-21-e13557-s008.jpg]

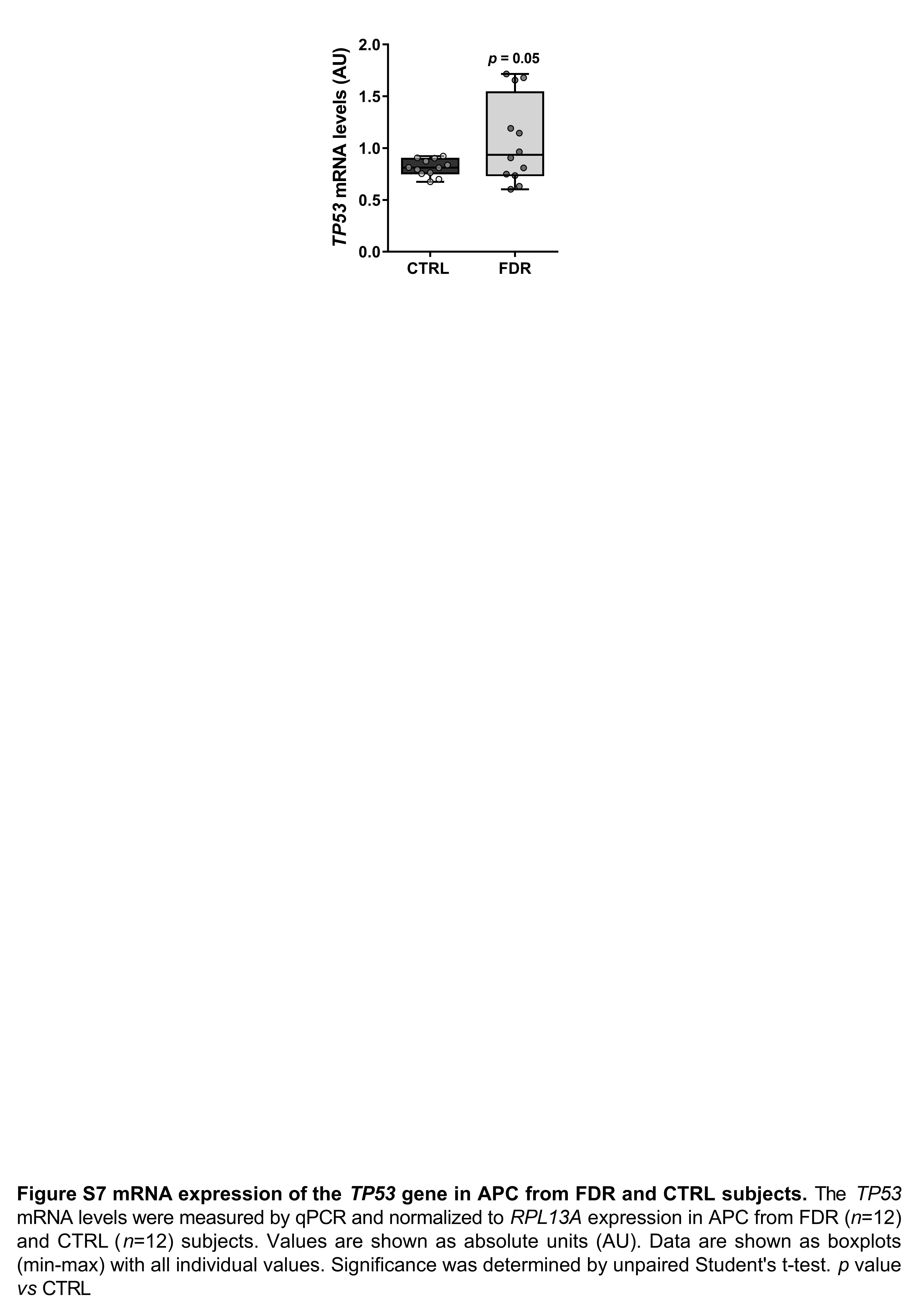

Supplement: Supplementary file 7 — Fig S7 [file ACEL-21-e13557-s020.jpg]

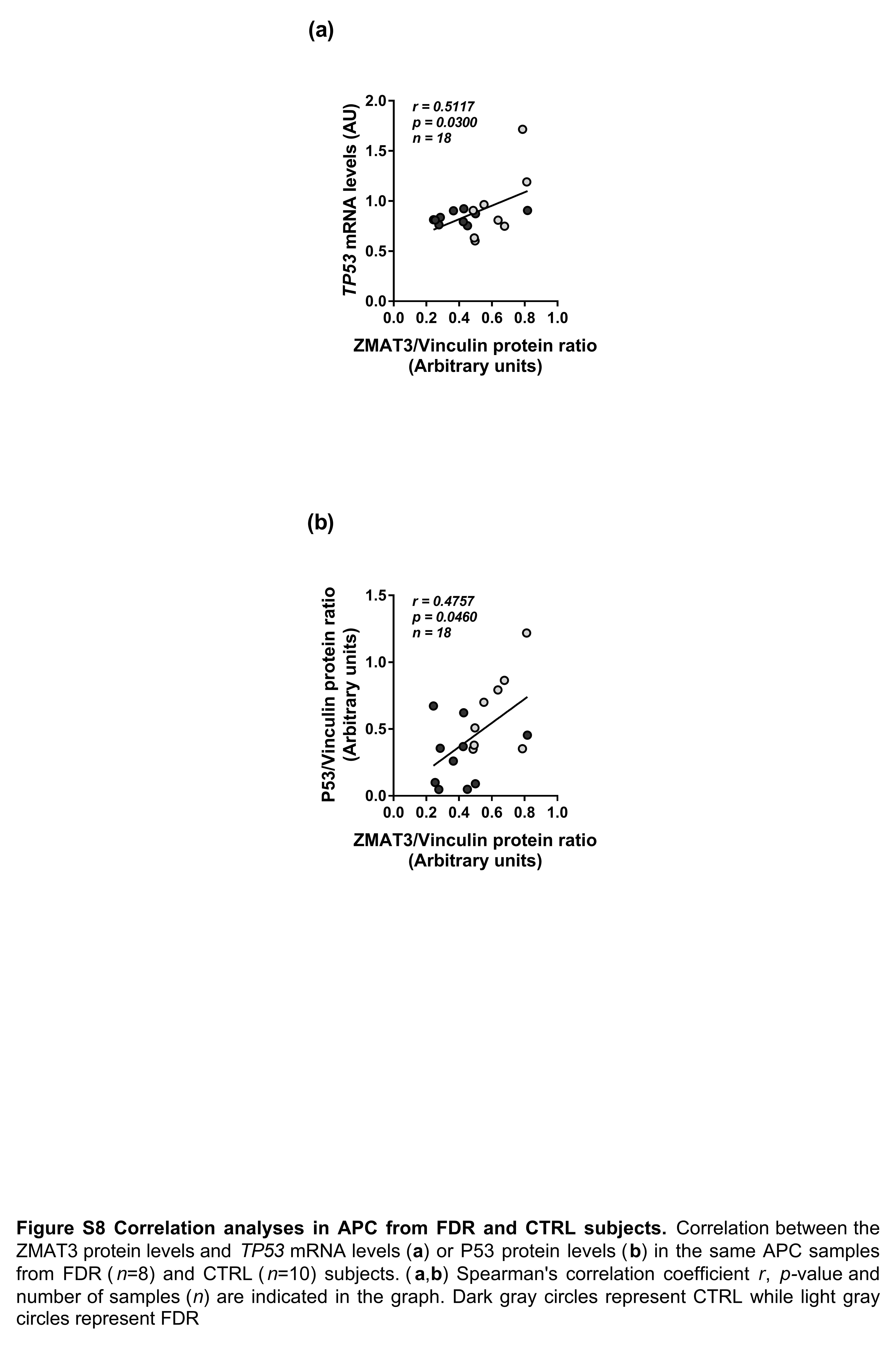

Supplement: Supplementary file 8 — Fig S8 [file ACEL-21-e13557-s018.jpg]

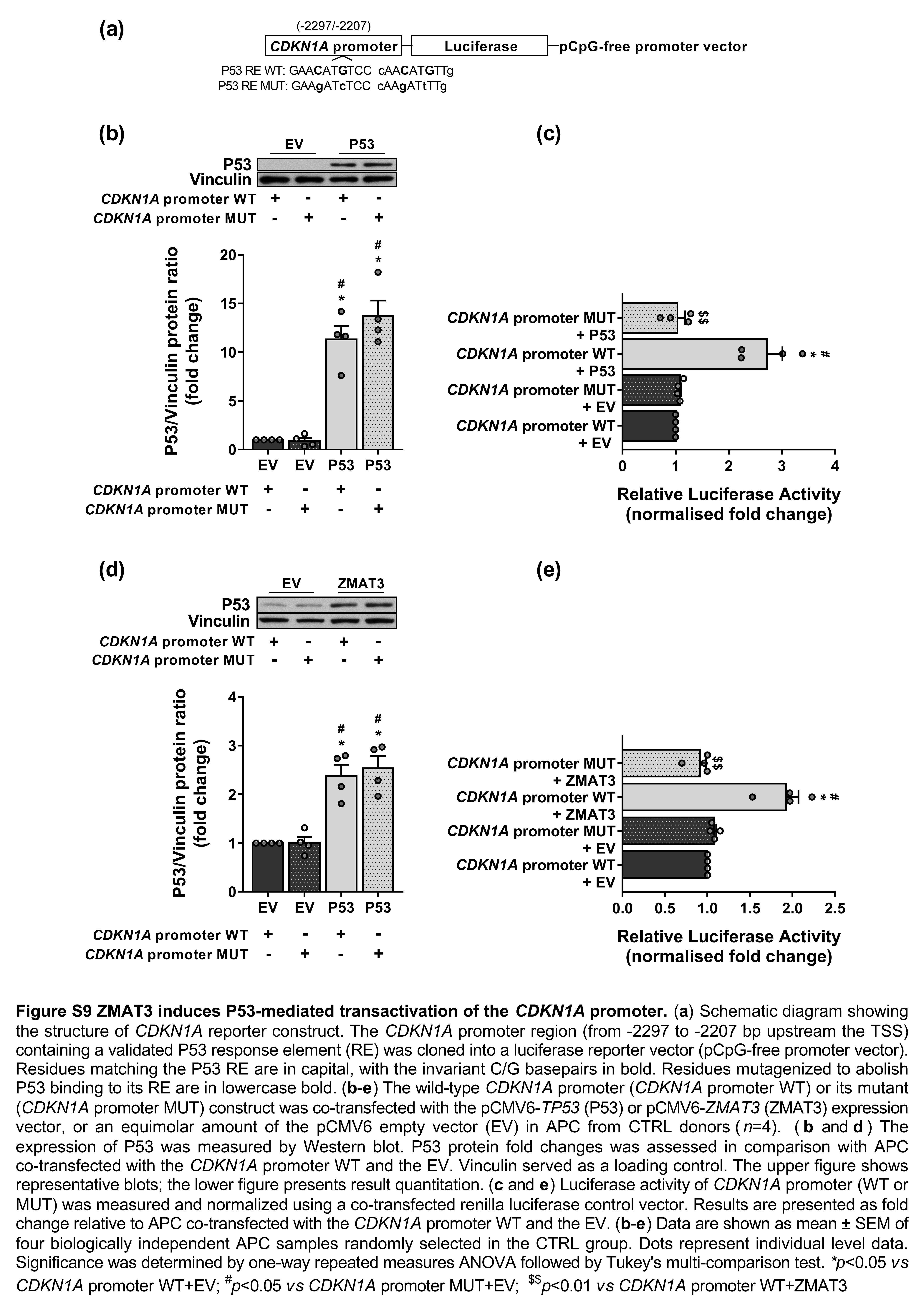

Supplement: Supplementary file 9 — Fig S9 [file ACEL-21-e13557-s007.jpg]

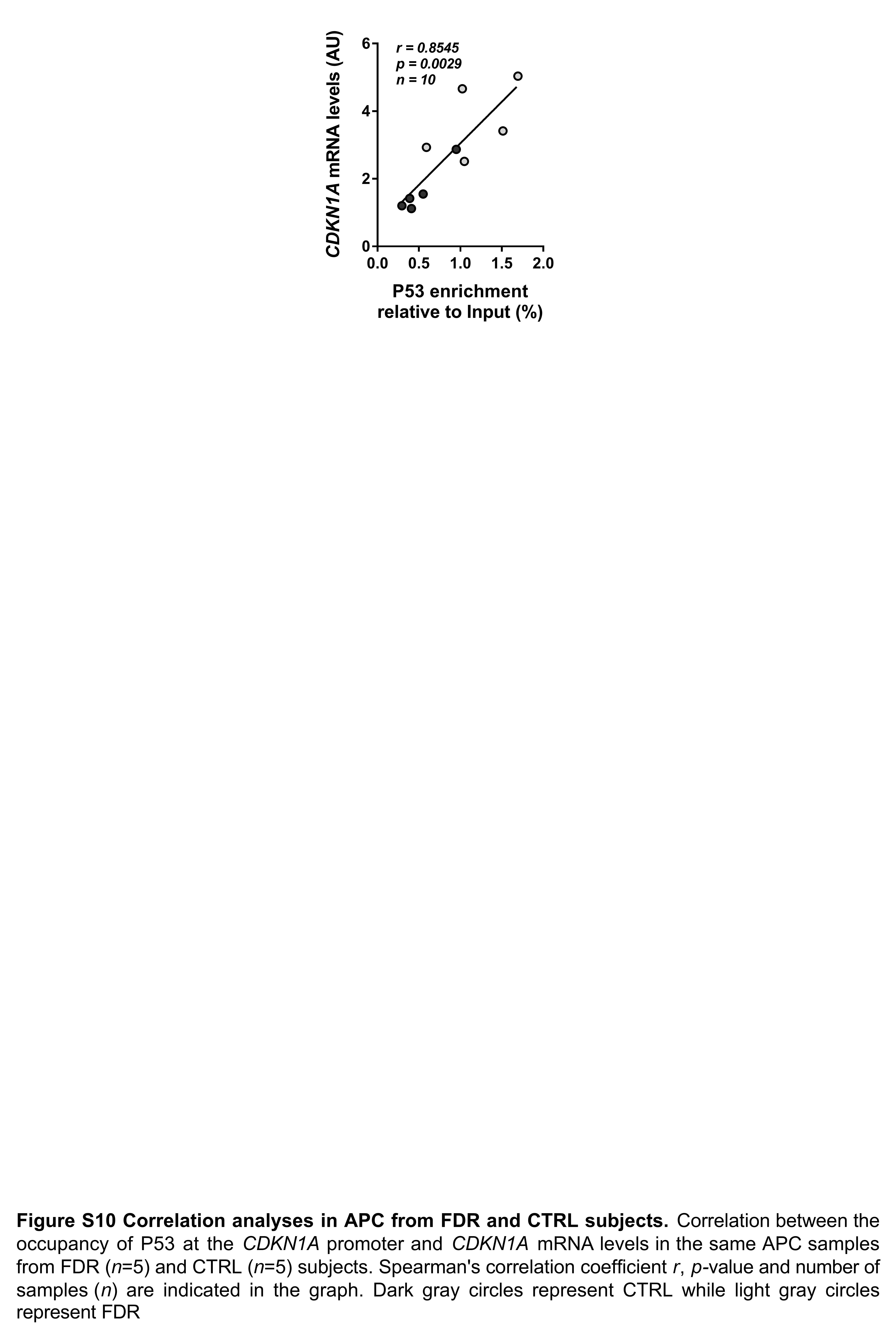

Supplement: Supplementary file 10 — Fig S10 [file ACEL-21-e13557-s010.jpg]

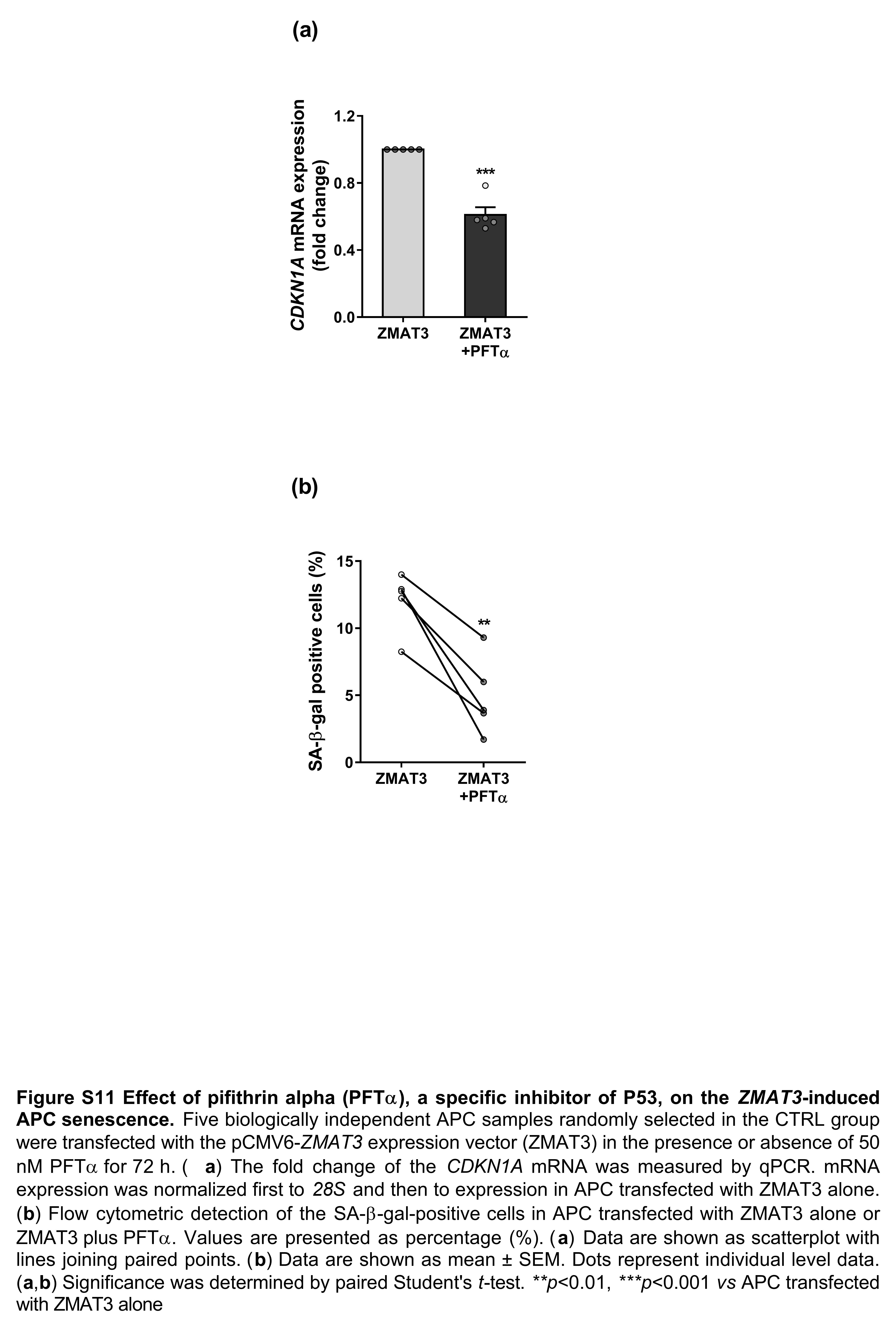

Supplement: Supplementary file 11 — Fig S11 [file ACEL-21-e13557-s019.jpg]

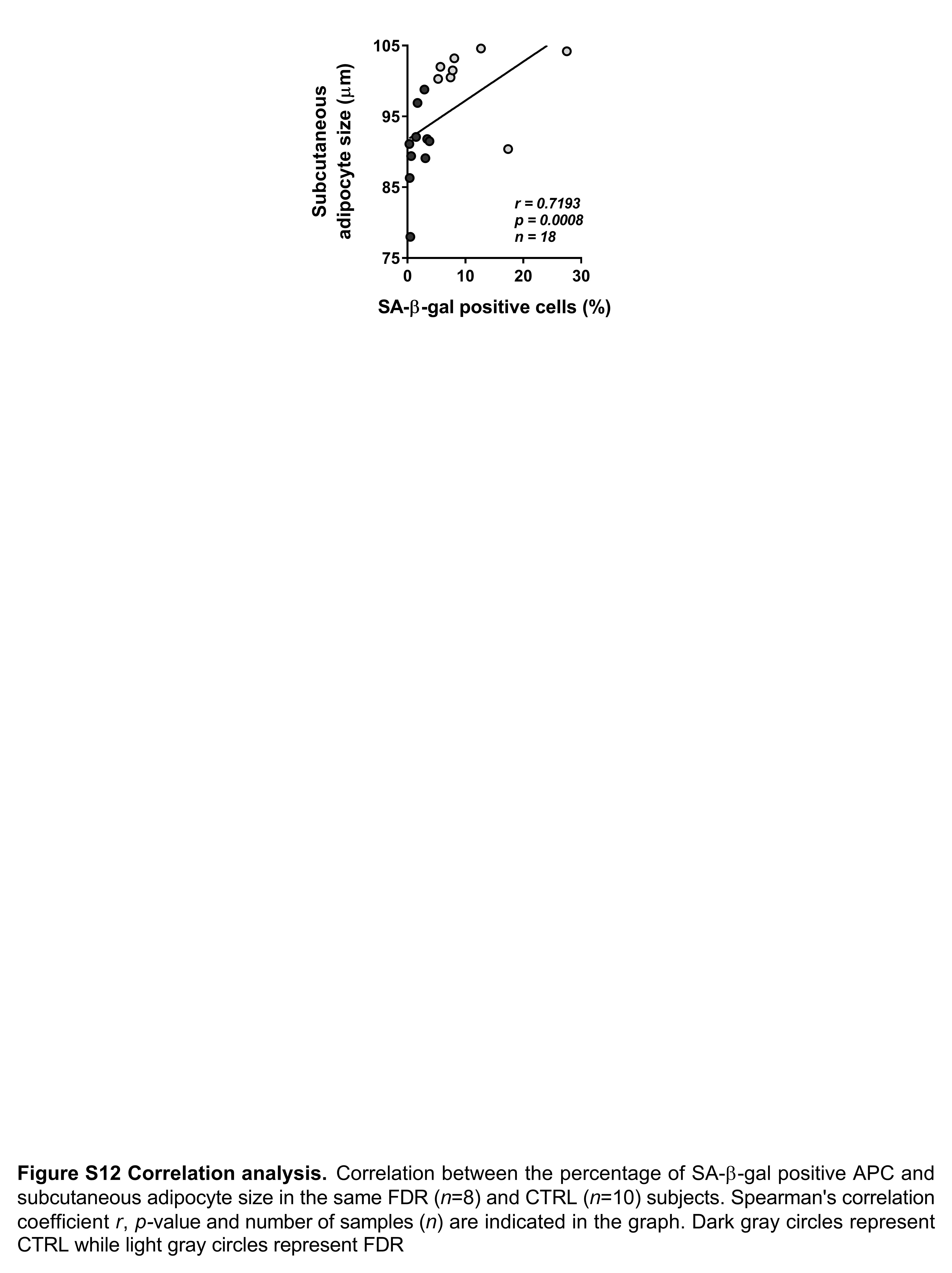

Supplement: Supplementary file 12 — Fig S12 [file ACEL-21-e13557-s002.jpg]

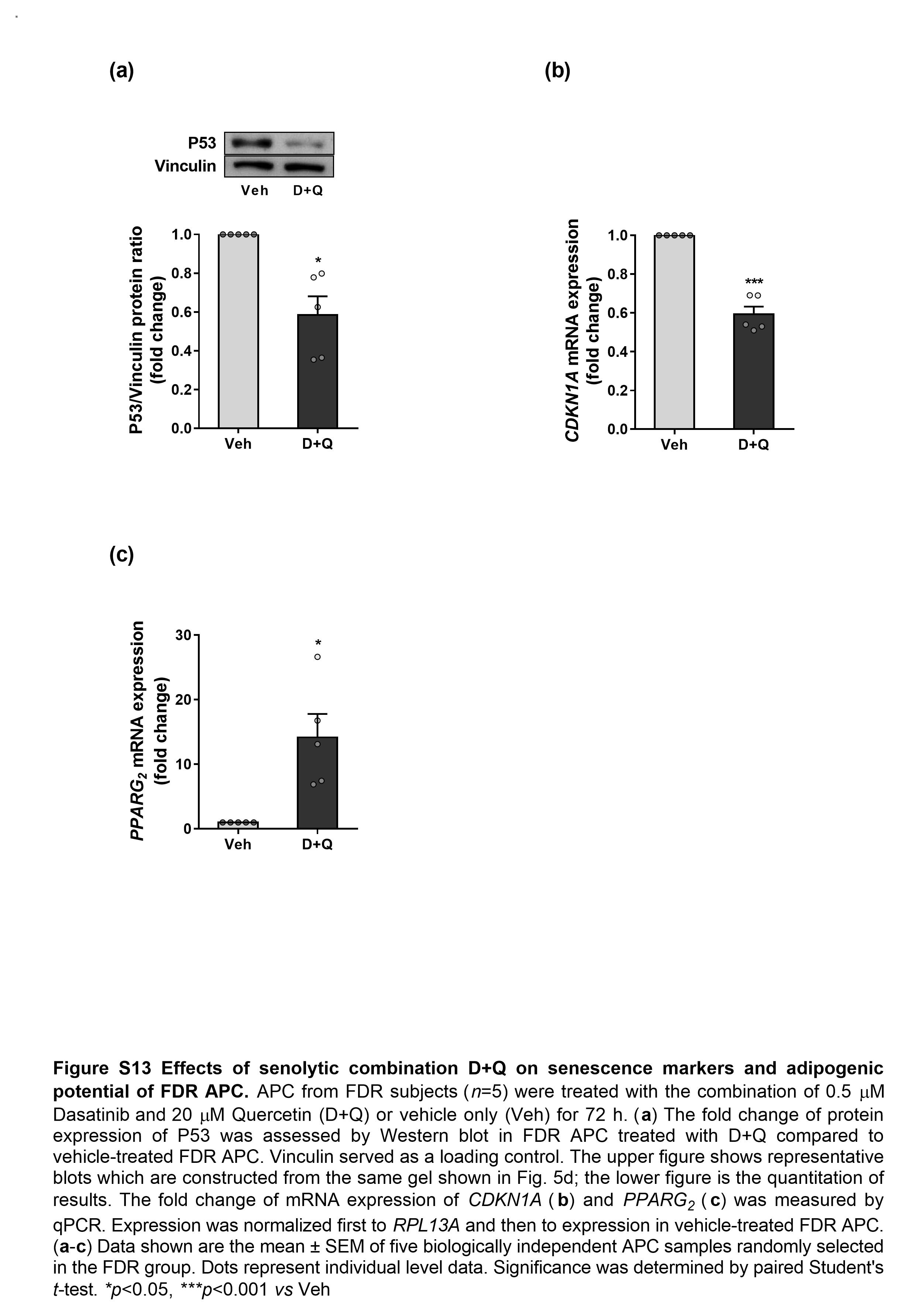

Supplement: Supplementary file 13 — Fig S13 [file ACEL-21-e13557-s009.jpg]

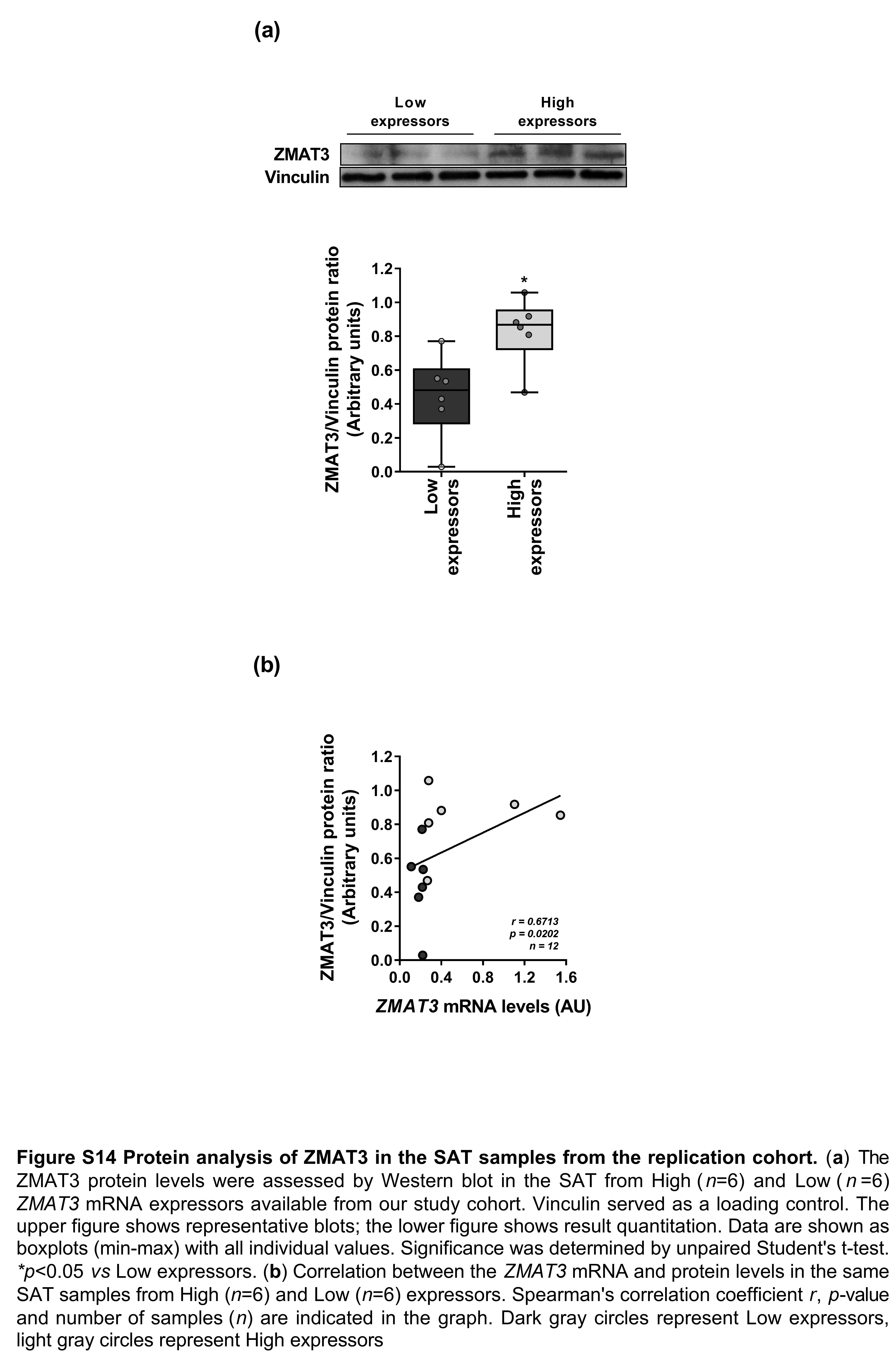

Supplement: Supplementary file 14 — Fig S14 [file ACEL-21-e13557-s016.jpg]
