# Supplementary material for: ZMAT3 hypomethylation contributes to early senescence of preadipocytes from healthy first‐degree relatives of type 2 diabetics
Source: Aging Cell. 2022 Feb 11;21(3):e13557. doi: 10.1111/acel.13557 (PMC8920444; doi:10.1111/acel.13557)
Supplement: Supplementary file 15 — Table S1 [file ACEL-21-e13557-s012.pdf]

Table S1: SASP factor protein levels in the media conditioned by *ZMAT3* overexpressing APC.

Variables	*ZMAT3*	*EV*
**IL6 (pg/ml/10^5 cells)**	*1509.0 ± 476.1**	*150.2 ± 147.9*
**MCP1 (pg/ml/10^5 cells)**	*186.6 ± 47.0**	*82.9 ± 31.8*
**RANTES (pg/ml/10^5 cells)**	*441.6 ± 170.5*	*124.1 ± 42.8*
**IL8 (pg/ml/10^5 cells)**	*1452.0 ± 646.3*	*80.5 ± 33.6*
**MIP1b (pg/ml/10^5 cells)**	*12.1 ± 2.3**	*4.2 ± 1.4*

SASP factor protein levels in the media conditioned by APC from CTRL donors (*n*=5) transfected with the pCMV6-*ZMAT3* expression vector (ZMAT3) or an equimolar amount of the pCMV6 empty vector (EV) were measured by a custom multiplex assay and normalized by cell number. All data shown are the mean ± SEM of five biologically independent APC samples randomly selected in the CTRL group. Significance was determined by paired Student's *t*-test. **p*<0.05 *vs* EV.

IL, interleukin; MCP1, monocyte chemotactic protein 1; RANTES, regulated on activation normal T-cell-expressed and -secreted; MIP1b, macrophage inflammatory protein 1 beta.
