# Supplementary material for: ZMAT3 hypomethylation contributes to early senescence of preadipocytes from healthy first‐degree relatives of type 2 diabetics
Source: Aging Cell. 2022 Feb 11;21(3):e13557. doi: 10.1111/acel.13557 (PMC8920444; doi:10.1111/acel.13557)
Supplement: Supplementary file 16 — Table S2 [file ACEL-21-e13557-s015.pdf]

Table S2. SASP factor protein levels in the media conditioned by FDR APC treated with Dasatinib plus Quercetin or vehicle only.

Variables	*D+Q*	*Veh*
**IL6 (pg/ml/10^5 cells)**	*93.6 ±20.6 ***	*1383.0 ± 215.0*
**MCP1 (pg/ml/10^5 cells)**	*12.2 ± 4.1***	*190.1 ± 28.3*
**RANTES (pg/ml/10^5 cells)**	*2.9 ± 0.3***	*6.9 ± 1.4*
**IL8 (pg/ml/10^5 cells)**	*53.1 ± 14.0***	*179.7 ± 20.3*
**MIP1b (pg/ml/10^5 cells)**	*0.4 ± 0.2$^{p=0.06}$*	*2.4 ± 0.3*

SASP factor protein levels in the media conditioned by FDR APC (*n*=5) treated with the combination of 0.5 μM Dasatinib and 20 μM Quercetin (D+Q) or vehicle only (Veh) for 72 h were measured by a custom multiplex assay and normalized by cell number. All data shown are the mean ± SEM of five biologically independent APC samples randomly selected in the FDR group. Significance was determined by paired Student's *t*-test. ***p*<0.01 *vs* Veh.

IL, interleukin; MCP1, monocyte chemotactic protein 1; RANTES, regulated on activation normal T-cell-expressed and -secreted; MIP1b, macrophage inflammatory protein 1 beta.
