# Supplementary material for: ZMAT3 hypomethylation contributes to early senescence of preadipocytes from healthy first‐degree relatives of type 2 diabetics
Source: Aging Cell. 2022 Feb 11;21(3):e13557. doi: 10.1111/acel.13557 (PMC8920444; doi:10.1111/acel.13557)
Supplement: Supplementary file 17 — Table S3 [file ACEL-21-e13557-s011.pdf]

Table S3. Characteristics of subjects in the discovery cohort stratified according to *ZMAT3* mRNA expression in subcutaneous adipose cells.

Phenotypes	*High ZMAT3 mRNA expression*	*Low ZMAT3 mRNA expression*	*p value*
**N**	14	15	
***ZMAT3* mRNA levels (REU)**	0.52 [0.39; 0.64]	0.21 [0.18; 0.31]	*<0.0001*
***TP53* mRNA levels (REU)**	0.53 [0.38; 0.91]	0.26 [0.19; 0.35]	*<0.0001*
**Age, years**	59.0 [32.8; 62.5]	45.0 [32.0; 47.0]	*0.0150*
**T2D: N (%)**	10 (71%)	0 (0%)	*<0.0001*
**BMI, Kg/m^2**	33.2 [30.5;35.0]	26.6 [23.1; 29.2]	*<0.0001*

Clinical study participants were stratified according to *ZMAT3* mRNA expression in subcutaneous adipose cells into two categories, low (L_{exp}) and high (H_{exp}) expression, defined by values below or above the median, respectively. Study participants are expressed as number (N). Data are shown as median [first quartile-Q1; third quartile-Q3]. Statistical differences between the two groups were tested using Mann Whitney test (continuous variables) or Fisher's exact test (categorical variable). *p* value *vs* L_{exp} participants.

REU, relative expression units; BMI, body mass index; T2D, Type 2 Diabetes.
