# Supplementary material for: ZMAT3 hypomethylation contributes to early senescence of preadipocytes from healthy first‐degree relatives of type 2 diabetics
Source: Aging Cell. 2022 Feb 11;21(3):e13557. doi: 10.1111/acel.13557 (PMC8920444; doi:10.1111/acel.13557)
Supplement: Supplementary file 18 — Table S4 [file ACEL-21-e13557-s001.pdf]

Table S4. Characteristics of subjects in the replication cohort stratified according to *ZMAT3* mRNA expression in subcutaneous adipose tissue.

Phenotypes	*High ZMAT3 mRNA expression*	*Low ZMAT3 mRNA expression*	*p value*
**N**	10	10	
***ZMAT3* mRNA levels (AU)**	0.32 [0.27; 0.65]	0.20 [0.15; 0.22]	*<0.0001*
***TP53* mRNA levels (AU)**	0.53 [0.45; 0.69]	0.35 [0.31; 0.38]	*<0.0001*
**Age, years**	53.0 [48.5; 58.5]	33.0 [21.0; 42.0]	*<0.0001*
**BMI, Kg/m^2**	29.6 [25.0;33.7]	27.6 [24.6; 30.8]	0.4030

Clinical study participants were stratified according to *ZMAT3* mRNA expression in subcutaneous adipose tissue into two categories, low (L_{exp}) and high (H_{exp}) expression, defined by values below or above the median, respectively. Study participants are expressed as number (N). Data are shown as median [first quartile-Q1; third quartile-Q3]. Statistical differences between the two groups were tested using Mann Whitney test. *p* value *vs* L_{exp} participants.

AU, absolute units; BMI, body mass index.
