# Supplementary material for: ZMAT3 hypomethylation contributes to early senescence of preadipocytes from healthy first‐degree relatives of type 2 diabetics
Source: Aging Cell. 2022 Feb 11;21(3):e13557. doi: 10.1111/acel.13557 (PMC8920444; doi:10.1111/acel.13557)
Supplement: Supplementary file 19 — Table S5 [file ACEL-21-e13557-s004.pdf]

Table S5. List of primers used in this study.

Technique	Gene/Region	Oligonucleotide sequences (5’ → 3’)
qPCR	*RPL13A*	F: CTTTCCGCTCGGCTGTTTTC<br>R: GCCTTACGTCTGCGGATCTT’
	CDKN1A	F: GCAGACCAGCATGACAGATTTC<br>R: ATGTAGAGCGGGCCTTTGAG
	LMNB1	F: GCCCAGATCAAGCTTCGAGA<br>R: GCTTCCAACTGGGCAATCTG
	ZMAT3	F: TATCGAAGGGAGGGGAGCAA<br>R: TTAAAGGAGCCCATCTGCGG
	TP53	F: CGCTTCGAGATGTTCCGAGA<br>R: CTTCAGGTGGCTGGAGTGAG
	28S	F: CCCAGTGCTCTGAATGTCAA<br>R: AGTGGGAATCTCGTTCATCC
	IL6	F: CAATGAGGAGACTTGCCTGGT<br>R: AGCTGCGCAGAATGAGATGA
	MCP1	F: CCCAAAGAAGCTGTGATCTTCA<br>R: TCTGGGGAAAGCTAGGGGAA
	$PPARG_2$	F: TCAGTGAATTACAGCAAACCC<br>R: AGTGTATCAGTGAAGGAATCGCT
Bisulfite sequencing	*ZMAT3* DMR	F: GGATTGTAGATAGAGTTT<br>R: TATTAAATACACCTCCCAAATA
Cloning	*ZMAT3* DMR forward	F: CGCCCTAGGCAGACAGAGTCTCGCTCACT<br>R: CGCGGATCCATGAGCTGTTGGGTACACCT
	ZMAT3 DMR reverse	F: CGCGGATCCCAGACAGAGTCTCGCTCACT<br>R: CGCCCTAGGATGAGCTGTTGGGTACACCT
	wild-type *CDKN1A* promoter	F: CGCCCTAGGAGCAGGCTGTGGCTCTGATT<br>R: CGCGGATCCCAAAATAGCCACCAGCCTCTTCT
	mutated *CDKN1A* promoter	F: CGCCCTAGGAGCAGGCTGTGGCTCTGATTGGC<br>TTTCTGGCCGTCAGGAA**G**AT**C**TCCCAA**G**AT**T**TTGAGCTC<br>TGGCATAGAAGAGGCTGGTGGCTATTTTGGGATCCCGC<br>R: GCGGGATCCCAAAATAGCCACCAGCCTCTTCTATG<br>CCAGAGCTCAAAATCTTGGGAGATCTTCCTGACGGCC<br>AGAAAGCCAATCAGAGCCACAGCCTGCTCCTAGGGCG

	ZMAT3 ORF	F: CTAGCTAGCATGATCCTCTTGCAACACGCCG R: CCGCTCGAGTACATATCCCAGATTCTCCATCTC
	TP53 ORF	F: CTAGCTAGCATGGAGGAGCCGCAGTC<br>R: CCGCTCGAGTCAGTCTGAGTCAGGCCCTT
**ChIP**	*CDKN1A* promoter	F: AGCAGGCTGTGGCTCTGATT<br>R: CAAAATAGCCACCAGCCTCTTCT
