# Supplementary material for: ZMAT3 hypomethylation contributes to early senescence of preadipocytes from healthy first‐degree relatives of type 2 diabetics
Source: Aging Cell. 2022 Feb 11;21(3):e13557. doi: 10.1111/acel.13557 (PMC8920444; doi:10.1111/acel.13557)
Supplement: Supplementary file 20 — Appendix S1 [file ACEL-21-e13557-s006.docx]

**Appendix S1**

**Study participants**

Twenty-four individuals were selected from the EUGENE2 consortium (Laakso et al., 2008). These subjects were healthy and non-obese with (*n*=12; FDR) or without (*n*=12; CTRL) one first-degree relative with T2D. Male/female in the study group was 1; mean age was 40.6 years (SEM:1.6 years); mean BMI was 24.9 Kg/m^2^ (SEM:0.4 Kg/m^2^). Detailed clinical characteristics of these individuals have been previously reported in (Laakso et al., 2008) and are synoptically presented in Table 2. Subjects who were FDR exhibited a significantly reduced insulin sensitivity and larger subcutaneous adipocytes when compared to CTRL subjects. No significant differences were shown between two groups regarding age, sex, BMI, and body fat percent. All of the enrolled subjects signed their informed consent and were subjected to abdominal subcutaneous adipose tissue (SAT) sampling from the paraumbilical region. The study protocol was approved by the Ethical Committee of the University of Gothenburg (ethical approval numbers S655-03 and T492-17) according to the Declaration of Helsinki.

**Isolation and culture of APC**

After harvesting, adipose precursor cells (APC) were isolated from SAT specimens as previously reported in (Gustafson et al., 2019). Minced adipose tissue was aseptically processed by incubation with collagenase (Roche Diagnostics) at 37 ºC for 45 min. The digest was filtered through nylon mesh with a pore size of 250 μm and collected in enzyme quenching media. The layer with floating adipocytes was removed and used for adipocyte size measurement as described in (Arner et al., 2011). The remaining media containing the APC fraction was centrifuged for 15 min at 1500 g at 20 ºC. The isolated APC were washed twice, and the erythrocytes were lysed with 155 mmol/l NH_4_Cl for 5 min before seeding APC. After 3 days, the inflammatory cells (CD14^+^/CD45^+^) and endothelial cells (CD31^+^) were removed from the APC fraction by immune magnetic separation (Miltenyi) as described in (Arner et al., 2011). The APC were then cultured with DMEM/F-12 medium (ThermoFisher Scientific) supplemented with 10% fetal bovine serum (FBS, ThermoFisher Scientific), 2 mmol/l glutamine, 100 U/ml penicillin and 100 μg/ml streptomycin (ThermoFisher Scientific). APC were finally expanded in culture for three passages to prepare for experimentations.

**Adipogenic differentiation of APC**

APC were grown and allowed to differentiate into mature adipocytes as described in (Gustafson et al., 2019). APC were induced to differentiate after 3 days of confluence (differentiation day 0) with a cocktail consisting of 850 nmol/l insulin, 10 μmol/l dexamethasone (Sigma-Aldrich), 0.5 mmol/l isobutylmethylxanthine (MP Biomedicals), 10 μmol/l rosiglitazone (Cayman Chemical) in DMEM/F12 supplemented with 3 % FBS, 2 mmol/l glutamine, and antibiotics. After 3 days, the medium was replaced to adipocyte medium consisting of 850 nmol/l insulin, 1 μmol/l dexamethasone, 1 μmol/l rosiglitazone in DMEM/F12 supplemented with 10 % FBS, 2 mmol/l glutamine, and antibiotics. The adipocyte medium was changed every 3 days throughout the differentiation period until day 15 (differentiation day 15). To examine lipid accumulation, differentiated APC were fixed with 4 % formaldehyde for 5 min at room temperature and stained with Oil Red O (Sigma-Aldrich) as previously reported in (Mirra et al., 2021).

**Flow cytometry analysis**

Flow cytometry was performed with a BD LSRFortessa Flow Cytometer (BD). For each experiment, 10,000 cells per APC sample were counted. Flow cytometric forward scatter (FSC-A) and side scatter (SSC-A) density plots were applied to analyze APC size and structure, respectively, as described (Ratushnyy et al., 2020). Senescence-associated beta-galactosidase (SA-β-gal) activity was assessed as indicated in (Debacq-Chainiaux et al., 2009). APC (3 x 10^4^ cells) were seeded in a 6-well plate. Cells at 70–80% confluence were treated with bafilomycin A1 (Sigma-Aldrich) for 1 hour to induce lysosomal alkalynization. This step was followed by 2 hours incubation with 5-dodecanoylaminofluorescein di-β-D-galactopyranoside (C_12_FDG, ThermoFisher Scientific). Once inside the cells, the C_12_FDG substrate is cleaved by SA-β-gal producing a green fluorescent product. SA-β-gal positive APC were quantified by flow cytometry. Cell cycle analysis was performed as reported in (Raciti et al., 2018). APC (3 x 10^4^ cells) were seeded in a 6-well plate and left until they had reached 70–80% confluency. Then, the cells were harvested, fixed in cold ethanol 70%, washed twice with cold PBS, and incubated in PBS containing 20 μg/mL PI and 1 mg/mL RNase A for 30 min at room temperature in the dark. DNA content of the PI-stained APC were analyzed by flow cytometry. The histogram of cell cycle distribution was generated from 10,000 events per APC sample.

**Primer sequences**

Sequences of all the used primers are shown in Table S5.

**Bisulphite sequencing**

Bisulphite treatment of genomic DNA (gDNA) extracted from APC by the AllPrep DNA/RNA Mini Kit (Qiagen) was carried out using the EZ DNA Methylation Kit (Zymo Research). Converted gDNA was amplified by PCR using specific primers for the *ZMAT3* DMR (*hg38_dna range=chr3:179032279-179033001*). Bisulphite sequencing was performed as previously reported in (Desiderio et al., 2019; Raciti et al., 2017). PCR products were cloned into the pGEM T-Easy vector (Promega) and 10 clones for sample were sequenced with an AB 3500 genetic analyzer (ThermoFisher Scientific). We first calculated the percentage of DNA methylation levels at all the 42 CpGs within the *ZMAT3* DMR in each clone and then averaged the DNA methylation level for 10 clones of the same sample.

**RNA isolation and qPCR**

Total RNA was extracted from APC using the AllPrep DNA/RNA Mini Kit (Qiagen). Reverse transcription of 1 μg of total RNA was performed using SuperScript III (ThermoFisher Scientific), following the manufacturer's instructions. qPCR reactions were run with SYBR Green PCR Master Mix (Bio-Rad) as previously described in (Longo et al., 2016; Nigro et al., 2019). Human *RPL13A* or *28S* were used as reference genes as mentioned in the corresponding figure legend. qPCR conditions were as follows: 95 °C for 30 s, 40 × (95 °C for 5 s and 60 °C for 30 s). All reactions were run in triplicate on a QuantStudio 7 Flex Real-Time PCR System (ThermoFisher Scientific).

**Western Blot**

Protein extracts were prepared in ice-cold RIPA buffer as previously described in (Pirone et al., 2019). Protein concentration was assessed using the protein assay based on Bradford’s method (Bio-Rad). Total cell extracts in equal amounts were separated by SDS-PAGE and blotted on nitrocellulose membrane (Millipore) as reported in (Ungaro et al., 2012). Upon incubation with primary antibodies against ZMAT3 (ab191536, Abcam), P53 (sc-126, Santa Cruz), or Vinculin (sc-73614, Santa Cruz), and secondary antibodies (Bio-Rad), immunoreactive bands were detected by an enhanced chemiluminescence kit (Bio-Rad) and quantified by the ImageJ software. Protein abundance was calculated after Vinculin normalization.

**Construction and functional analysis of luciferase reporter vectors**

The intronic *ZMAT3* DMR (*hg38_dna range=chr3:179032279-179033001*) was amplified by PCR and cloned into the CpG-free promoter firefly luciferase reporter vector (InvivoGen) in both forward and reverse orientations (Bakshi et al., 2018). Luciferase assay was performed as described in (Desiderio et al., 2019). Constructs were amplified in E. coli GT115 cells (InvivoGen). *In vitro* methylation was carried out using the M.SssI CpG methyltransferase (New England Biolabs) and S-adenosylmethionine (SAM; New England Biolabs), following manufacturer’s instructions. Un-methylated constructs were treated as the methylated construct, including application of SAM, but in the absence of *M.SssI* (mock-treated). *In vitro* methylation was confirmed by resistance to *HhaI* or *HpaII* (New England Biolabs) digestion. Transfection with an equimolar amount of the mock-treated empty vector was used to control for background firefly luciferase activity. Firefly luciferase activity of each transfection was normalized against renilla luciferase activity (Promega).

The wild-type *CDKN1A* promoter region (*hg38_dna range=chr6: 36676412-36676502*) containing the P53 response element (RE) from -2281 to -2261 bp upstream the TSS (el-Deiry et al., 1993; Laptenko et al., 2011) was amplified by PCR and cloned into the CpG-free promoter firefly luciferase reporter vector (InvivoGen). Complementary oligonucleotides corresponding the above-mentioned *CDKN1A* promoter region were synthesised by Sigma-Aldrich to incorporate the desired point mutations of the invariant G/C basepairs within the P53 RE to prevent P53 from binding to this nucleotide sequence (Kaeser & Iggo, 2004). These oligonucleotides were annealed *in vitro* and then cloned into the CpG-free promoter firefly luciferase reporter vector (InvivoGen). The wild-type or mutagenized *CDKN1A* reporter construct was transfected in APC from CTRL subjects in the presence of the pCMV6-*ZMAT3* or pCMV6-*TP53* expression vector, or an equimolar amount of the pCMV6 empty vector. Co-transfection of the wild-type *CDKN1A* reporter construct with the pCMV6 empty vector was used to control for the basal *CDKN1A* promoter activity. Firefly luciferase activity of each transfection was normalized against renilla luciferase activity (Promega).

All final constructs were validated by sequencing. Transfections were carried out by Lipofectamine 3000 reagent (ThermoFisher Scientific) in serum-free Opti-MEM media (ThermoFisher Scientific), following manufacturer's instructions. Luciferase activities were measured by a dual‐luciferase reporter system and a GloMax Luminometer (Promega). All luciferase assays were performed at least three times.

**Construction and transfection of *ZMAT3* expression vector**

The *ZMAT3* expression vector was engineered using the pCMV6-Entry mammalian expression plasmid (Origene). The complete ORF of *ZMAT3* (RefSeq NM_022470.4) was amplified from full-length cDNA by PCR. The *ZMAT3* ORF was cloned into the pCMV6 plasmid digested with NheI and XhoI (New England BioLabs) to generate the recombinant vector pCMV6-*ZMAT3*. The final construct was verified by sequencing. APC from CTRL donors were transfected with the pCMV6-*ZMAT3* expression vector or an equimolar amount of the pCMV6 empty vector using Lipofectamine 3000 reagent (ThermoFisher Scientific) in serum-free Opti-MEM media (ThermoFisher Scientific), following manufacturer's instructions. Senescence markers were evaluated 3 days after transfection.

**Construction and transfection of *TP53* expression vector**

The *TP53* expression vector was engineered using the pCMV6-Entry mammalian expression plasmid (Origene). The complete ORF of *TP53* (RefSeq NM_000546) was amplified from full-length cDNA by PCR. The *TP3* ORF was cloned into the pCMV6 plasmid digested with NheI and XhoI (New England BioLabs) to generate the recombinant vector pCMV6-*TP53*. The final construct was verified by sequencing. APC from CTRL donors were transfected with the pCMV6-*TP53* expression vector or an equimolar amount of the pCMV6 empty vector using Lipofectamine 3000 reagent in serum-free Opti-MEM media and following manufacturer's instructions.

**Multiplex SASP protein analysis**

Conditioned media (CM) were prepared by pre-washing APC cultures with PBS, then exposing them to serum-free DMEM/F-12 medium (ThermoFisher Scientific) for 24 hours. CM were centrifuged at 14,000 g to remove debris and stored at -80 °C for subsequent analysis. Bioplex Multiplex human cytokine and chemokine assays (Bio-Rad) were used to quantify SASP factors in CM as specified by the supplier.

**Chromatin immunoprecipitation (ChIP)**

ChIP experiments were performed by True MicroChIP kit (Diagenode), following manufacturer's instructions. APC from CTRL donors transfected with the pCMV6-*ZMAT3* expression vector or an equimolar amount of the pCMV6 empty vector, as well as APC from FDR and CTRL subjects, were cross-linked with 1 % formaldehyde for 10 min at room temperature and then quenched with 125 mmol/l glycine for 5 min. The samples were sonicated for chromatin shearing using a Bioruptor (Diagenode). After centrifugation, the supernatants were diluted according to the manufacturer’s instructions. The samples of sheared chromatin were divided into Input, P53-IP, and IgG control aliquots. At the latter two aliquots were added 2 µg of anti-P53 monoclonal antibody (sc-126, Santa Cruz) or mouse IgG (sc-2025, Santa Cruz) as a negative control (IgG control), respectively, and incubated for 16 hours at 4 °C with rotation. Protein G-coated magnetic beads (ThermoFisher Scientific) were added to each sample, which was then incubated for 2 hours at 4 °C. The immunoprecipitated DNA was eluted from coated magnetic beads and subsequently purified with QIAquick PCR Purification Kit (Qiagen). The DNA samples (Input, P53-IP, and IgG control) were subjected to qPCR amplification using primers flanking the region of interest within the *CDKN1A* promoter.

**Senescence induction**

To induce senescence, APC obtained from CTRL donors were exposed to hydrogen peroxide (H_2_O_2_; Sigma-Aldrich) or 5-azacytidine (5-AZA; Sigma-Aldrich). H_2_O_2_ was dissolved in culture medium at a concentration of 200 µM and APC were treated as described in (Wang et al., 2013). 5‐AZA was dissolved in culture medium at a concentration of 10 µM and APC were treated for 72 hours.

**RNA interfering**

siRNA targeting *ZMAT3* (siRNA*^ZMAT3^*) and scrambled siRNA as negative control (siRNA*^C^*) were purchased from ThermoFisher Scientific. APC from CTRL donors were transfected with 25 pmol of siRNA*^ZMAT3^* or siRNA*^C^* using Lipofectamine 3000 reagent (ThermoFisher Scientific) in serum-free Opti-MEM media (ThermoFisher Scientific), following manufacturer's instructions. After 24 hours from the transfection, the cells were treated for 72 hours with or without 10 µM 5-AZA in the absence or presence of either siRNA*^ZMAT3^* or siRNA*^C^*.

**PFTα treatment**

PFTα was purchased from Santa Cruz (sc-45050). PFTα was dissolved in DMSO to generate stock solution (50 mM). Then, it was diluted in DMEM/F12 medium to a final concentration of 50 nM. APC from CTRL donors were transfected with the pCMV6-*ZMAT3* vector in the presence or absence of 50 nM PFTα for 72 hours.

**Senolytic treatment**

Dasatinib (D) and Quercetin (Q) were purchased from Sigma-Aldrich. D and Q were dissolved in DMSO to generate stock solutions (100 mM and 10 mM, respectively). Then, D plus Q was diluted in DMEM/F12 medium to get the indicated final concentration. APC from FDR donors were exposed to 0.5 μM D plus 20 μM Q or vehicle only (DMSO) for 3 days as reported in (Zhu et al., 2015).

**SAT bioptical samples**

**Discovery cohort -** Human abdominal SAT samples were obtained in the fasting state by needle biopsy from *n*=29 subjects aged 26 to 67. This group included both T2D individuals (*n*=10) and subjects who featured normal glucose tolerance (*n*=19). Their clinical characteristics have been previously reported (Gustafson et al., 2019). Prior to sample collection, all subjects provided informed consent in full compliance with and strict adherence to the guidelines approved by the Ethical Committee of the University of Gothenburg in agreement with the Declaration of Helsinki. Whole tissue was used for adipose cell isolation (Gustafson et al., 2019). The isolated adipose cells were directly processed for RNA extraction. Gene expression was analysed with the Quant Studio6 sequence detection system (ThermoFisher Scientific). mRNA results were first normalized to *28S* and then normalized to expression levels in one individual (=1). Values are presented as relative expression units (REU).

**Replication cohort -** Human SAT samples were obtained from women (*n*=20; age 18–61 years; BMI 22.0–43.3) undergoing surgical mammary reduction. Sample size was calculated by using the G*Power 3.1.9.2 software (Heinrich-Heine-Universität Düsseldorf, Germany). A sample size of 20 participants, *n*=10 for group, achieved 95% power to detect a difference of 0.3692 between the null hypothesis that both group means are equal to 0.2299 and the alternative hypothesis that the mean of group 2 is different and equal to 0.5491, considering a two-sample t test (two tails), a significance level (α) of 0.01, an estimated group standard deviations of 0.0908 and 0.1843, and an allocation ratio N2/N1 = 1. For calculations, values of the *ZMAT3* mRNA (mean ± SD) in high- and low-expressor subjects from our discovery cohort were applied. All of the women were otherwise healthy, with no metabolic or endocrine diseases. Their characteristics have been previously described (D’Esposito et al., 2012). Before the surgical procedure, each study participant provided informed consent. This procedure was approved by the ethical committee of the University of Naples. The mammary adipose tissue specimens were directly processed for RNA extraction and gene expression analysis. Among them, twelve samples were available for protein extraction and expression analysis.

**Statistical analysis**

Data are presented according to proposed guidelines for basic science data visualization (Weissgerber et al., 2017). Biological replicates were collected from different samples, each isolated from different human specimens. The number of independent biological replicates (*n*) used in each experiment was indicated in the figure. Statistical analysis was performed with GraphPad Prism 6.0 software (GraphPad Software Inc) and R statistical platform. Normal distribution of continuous variables was tested using the Shapiro-Wilk test. Normally distributed data were compared between groups by unpaired Student's *t-*test (two-tailed). Within-group comparisons between matched samples were performed using paired two-tailed Student *t*-test or one-way repeated measures ANOVA followed by Tukey's multi-comparison test, as appropriate. Not normally distributed data were compared between groups by Mann Whitney test (two-tailed). The correlation between quantitative variables was tested using Spearman's rank correlation test. The association between age or *TP53* mRNA levels and *ZMAT3* expression in both subcutaneous adipose cells and SAT was tested by multiple regression analysis adjusting for BMI. BMI was fixed as covariate to account for the potential confounding effect of the association between this variable and cellular senescence and mRNA expression in human SAT. *p-*value ≤0.05 was considered statistically significant

**Supplementary reference**

Bakshi, A., Bretz, C. L., Cain, T. L., & Kim, J. (2018). Intergenic and intronic DNA hypomethylated regions as putative regulators of imprinted domains. Epigenomics, 10(4), 445–461. <https://doi.org/10.2217/epi-2017-0125>

Kaeser, M. D., & Iggo, R. D. (2004). Promoter-specific p53-dependent histone acetylation following DNA damage. Oncogene, 23(22), 4007–4013. <https://doi.org/10.1038/sj.onc.1207536>

Wang, Z., Wei, D., & Xiao, H. (2013). Methods of cellular senescence induction using oxidative stress. Methods in molecular biology (Clifton, N.J.), 1048, 135–144. <https://doi.org/10.1007/978-1-62703-556-9_11>
